# Treatment of lung diseases *via* nanoparticles and nanorobots: Are these viable alternatives to overcome current treatments?

**DOI:** 10.1016/j.mtbio.2025.101616

**Published:** 2025-02-26

**Authors:** Meekha George, Rabah Boukherroub, Amitav Sanyal, Sabine Szunerits

**Affiliations:** a Laboratory for Life Sciences and Technology (LiST), Faculty of Medicine and Dentistry, Danube Private University (DPU), Viktor-Kaplan-Straße 2, Geb. E, 2700, Wiener Neustadt, Austria; b Univ. Lille, CNRS, Univ. Polytechnique, Hauts-de-France, UMR 8520 - IEMN, F-59000, Lille, France; c Department of Chemistry, Bogazici University, Bebek, 34342, Istanbul, Turkey

**Keywords:** Nanoparticles, Biohybrid nanorobots, Nanomedicine, Lung diseases, Drug delivery

## Abstract

**Challenges:**

Respiratory diseases remain challenging to treat, with current efforts primarily focused on managing symptoms rather than maintaining overall lung health. Traditional treatment methods, such as oral or parenteral administration of antiviral, antibacterial, and anti-inflammatory drugs, face limitations. These include difficulty in delivering therapeutic agents to pathogens residing deep in the airways and the risk of severe side effects due to high systemic drug concentrations. The growing threat of drug-resistant pathogens further complicates infection management.

**Advancements:**

The lung's large surface area offers an attractive target for inhalation-based drug delivery. Nanoparticles (NP) enable uniform and sustained drug distribution across the alveolar network, overcoming challenges posed by complex lung anatomy. Recent breakthroughs in nanorobots (NR) have demonstrated precise navigation through biological environments, delivering therapies directly to affected lung areas with enhanced accuracy. Nanotechnology has also shown promise in treating lung cancer, with nanoparticles engineered to overcome biological barriers, improve drug solubility, and enable controlled drug release.

**Future scope:**

This review explores the progress of NP and NR in addressing challenges in pulmonary drug delivery. These innovations allow targeted delivery of nucleic acids, drugs, or peptides to the pulmonary epithelium with unprecedented accuracy, offering significant potential for improving therapeutic effectiveness in respiratory disorders.

## Highlights

• The therapeutic potentials of nanoparticles and nanorobots against lung diseases are reviewed.
• The role of biohybrid nanorobots in overcoming barriers for treating complex lung infections and cancer is analyzed.
• Inhalable nanomedicines for localized treatment are evaluated.

## Graphical abstract

**Figure:**
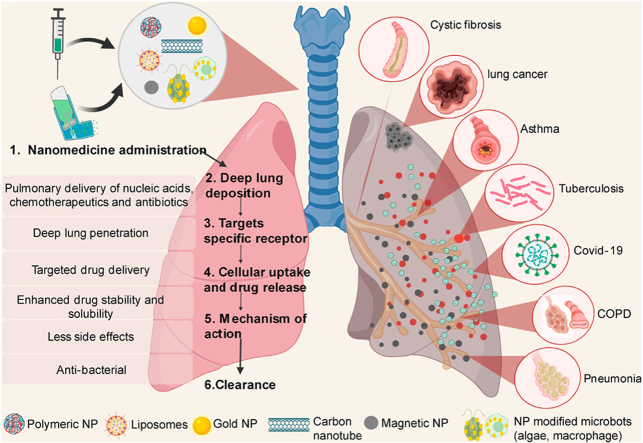

## Abbreviation

AM: Alveolar macrophages
bioNR: Biohybrid nanorobots
CF: Cystic fibrosis
COPD: Chronic obstructive pulmonary disease
Dox: Doxorubicin
EPR: Enhanced permeability and retention
FDA: Food and drug administration
MSNP: Mesoporous silica nanoparticles
NP: Nanoparticles
NR: Nanorobots
NSCLC: Non-small cell lung cancer
RBC: Red blood cells
SCLC: Small cell lung cancer
TB: Tuberculosis

## 1 Introduction

Respiratory disorders stand on challenging ground, affecting millions globally and posing significant concerns for both patients and healthcare professionals alike [1]. They encompass a wide range of conditions, including bacterial and viral pneumonia, chronic obstructive pulmonary disease (COPD), pneumonia, tuberculosis (TB), cystic fibrosis (CF) and lung cancer [2,3]. More recently, this category has broadened to include emerging infectious diseases like Middle East Respiratory Syndrome Coronavirus (MERS-CoV) [4], influenza A/H1N1 [5], and SARS-CoV-2 [6]. These different respiratory diseases can be grouped into four major categories.

(1) *Inflammatory and chronic respiratory disorders*: Several conditions fall under this category. COPD ranks as the third-leading cause of mortality worldwide, claiming the lives of 3.2 million individuals annually and contributing to a staggering 81.7 % of all fatalities attributed to chronic respiratory diseases [7,8]. Asthma, although not fatal in most cases, impacts the quality of life for millions through persistent inflammation and airway hyper-responsiveness [9]. Management includes bronchodilators (beta-agonists, anticholinergics), and inhaled corticosteroids, in addition to simple lifestyle modifications such as smoking cessation [10].
(2) *Infectious diseases*: This category includes bacterial and viral pneumonia, TB, and more recent challenges like SARS-CoV-2. Pneumonia emerges as a primary cause of death among children under 5 years old beyond the neonatal stage, as well as among adults over 65 years old [7,11]. TB, poised to be the foremost infectious cause of death, faced formidable competition from the ongoing COVID-19 pandemic, which accounted for a significant portion of global mortality [7,12]. The treatment of bacterial pneumonia caused by *Staphylococcus aureus*, *Klebsiella pneumoniae, Pseudomonas aeruginosa*, and/or *Acinetobacter baumannii*, typically involves the administration of broad-spectrum antibiotics. For *S. aureus*, treatment depends on methicillin resistance, with vancomycin used for methicillin-resistant *S. aureus* (MRSA). Gram-negative pathogens are often treated with newer beta-lactam/beta-lactamase inhibitor combinations such as ceftazidime-avibactam, meropenem-vaborbactam, or ceftolozane-tazobactam [13]. TB treatment relies also on a combination of antibiotics (e.g. isoniazid, rifampicin, pyrazinamide, and ethambutol) to be taken over several months to ensure complete eradication of the *Mycobacterium tuberculosis* bacteria [14]. In the case of viral pneumonia, although specific antiviral agents do exist, the cornerstone of treatment remains supportive care, including oxygen therapy and fluids [15]. The same is valid for COVID-19, which can be managed through a combination of antiviral drugs (e.g. remdesivir and nirmatrelvir), treatment with corticosteroids like dexamethasone to reduce inflammation, as well as simple oxygen therapy, and in severe cases, mechanical ventilation [16].
(3) *Neoplastic diseases*: Neoplastic diseases of the respiratory system represent a significant global health challenge due to their aggressive nature and limited survival outcomes. Lung cancer, notorious for its lethality, encompasses two major types: non-small cell lung cancer (NSCLC) and small cell lung cancer (SCLC). NSCLC accounts for approximately 85 % of cases and typically progresses more slowly, with adenocarcinoma being the most common subtype. In contrast, SCLC is highly aggressive and associated with rapid tumor growth and early metastasis, making its prognosis particularly poor. Lung cancer, in general, exhibits dismal five-year survival rates ranging from merely 10 %–20 %, even in developed nations [17,18]. The current treatments vary depending on the stage and type of the cancer. For early-stage NSCLC, surgical resection (e.g. lobectomy, pneumonectomy, segmentectomy) is utilized, with minimally invasive techniques like video-assisted thoracoscopic surgery (VATS) and robotic-assisted thoracic surgery (RATS), offering quicker recovery [19]. Radiation therapies (e.g. stereotactic body radiotherapy, intensity-modulated radiation therapy) provide precise tumor targeting while minimizing damage to healthy tissues [20,21], while follow-up chemotherapy with platinum-based drugs (e.g. oxaliplatin, cisplatin, carboplatin) remains essential [22]. Interestingly, recent findings from a phase II clinical trial on the combination of Nab-paclitaxel and gemcitabine for patients with advanced non-squamous NSCLC, following platinum-based therapy, suggest that multi-agent chemotherapy does not necessarily result in greater efficacy [23]. Therapies targeting epidermal growth factor receptor (EGFR), such as erlotinib, gefitinib, and osimertinib (EGFR inhibitors), have been found to be more adapted for *EGFR-mutated* NSCLC patients [24].
(4) *Genetic disorder*: Unlike other conditions that often develop later in life, CF impacts patients from birth, caused by mutation in the CFTR gene which impairs the normal clearance of mucus from the lungs and requires lifelong management [25]. The timely treatment of these lung disorders is essential to improve the life conditions of patients. The Food and Drug Administration's (FDA) approval of three-concept therapy based on elexacaftor/tezacaftor/ivacaftor marked a significant milestone in CF treatment. This triple combination therapy is effective for patients aged 12 and older who have at least one F508del mutation, the most common mutation causing CF and affecting about 90 % of CF patients [26].

Issues such as drug resistance in TB, incorrect inhaler use in COPD, certain CFTR mutations that do not respond to CFTR modulators in CF, and immune-related side effects in lung cancer therapies further complicate their management [[27], [28], [29]]. It has to be kept in mind that chronic respiratory diseases, such as COPD or asthma, are not curable with various forms of treatment that help open the air passages and improve shortness of breath available. For COPD or asthma, a 5-day treatment course is typically recommended, as prolonged use of steroid tablets may lead to undesirable side effects, including weight gain, mood swings, and weakened bones (osteoporosis) [30]. In the case of infectious lung diseases, treatment with antibiotics such as vancomycin and others may not be effective due to antibacterial/drug resistance. It is also often associated with several side effects, including nephrotoxicity, ototoxicity, and infusion-related reactions, which limit its long-term use and may require careful monitoring of renal function during therapy [31]. Lung cancer patients may experience side effects including hair loss, and feelings of weakness next to long-term neuropathy and hearing loss due to often high doses of chemotherapeutics required. In all cases of lung disease treatments, challenges persist in improving therapeutic efficacy, minimizing side effects, and improving patient adherence and compliance. Patient compliance is a major concern in the treatment of lung diseases, as many therapies involve long-term use or complex regimens. To improve adherence, strategies such as developing more convenient dosing schedules, utilizing combined inhaled medications, and implementing comprehensive patient education programs are essential. Reducing side effects and enhancing treatment safety profiles also contribute to better patient willingness to follow prescribed therapies. Healthcare providers can further improve compliance through continuous monitoring, clear communication, and personalized treatment plans. Addressing these factors can lead to improved treatment outcomes, fewer hospitalizations, and enhanced quality of life for patients managing chronic respiratory conditions. Overcoming these challenges requires continuous research to develop innovative drug delivery systems that enhance treatment outcomes while minimizing adverse effects [[27], [28], [29]]. The application of nanotechnology in medicine has garnered significant attention to boost drug efficiency, while limiting adverse reactions with hope also for the treatment of lung diseases [32,33].

## 2 Nanoparticles and nanorobots for the treatment of lung diseases

Nanoparticles (NP) are defined as particles with dimensions ranging from 1 to 100 nm. They are typically passive structures made from organic or inorganic materials, designed to carry drugs, imaging agents, or other therapeutic payloads. Due to their small size, NP exhibit unique physicochemical properties compared to bulk materials, such as an increased surface area-to-volume ratio, enhanced reactivity, and the ability to interact with biological systems at the molecular level [34,35]. Encapsulating poorly soluble drugs in hydrophobic particle cavities addresses solubility issues, but also ensures their biological activity by protecting them from degradation [36]. Additionally, the ability to target tumors and pathogens more precisely reduces harm to healthy tissues, thereby enhancing both the efficacy and safety of the treatment [37,38].

Nanorobots (NR), on the other hand, are more complex, engineered nanosystems that combine the features of NP with additional functionalities [39,40]. They can be defined as artificially fabricated nanoscale devices designed to perform specific tasks at the molecular or cellular level. The diminutive size of NR, typically ranging from the micro to nanometre size, enables direct cell interaction and penetration, facilitating precise, targeted delivery of therapeutic agents. NR often incorporate elements that allow for active movement, sensing capabilities, and programmable responses to environmental stimuli. These NR are equipped with an engine capable of converting various energy sources into mechanical force to perform therapeutic or diagnostic tasks [[41], [42], [43]]. The primary distinction between the nanostructures NR and NP lies thus in NR possessing an active power system that allows them to move and perform tasks, whereas nanocarriers are passive structures that rely on external forces to move or interact with their environment [44]. They may include biohybrid systems that combine synthetic materials with biological components, such as bacteria or cell fragments referred to as biohybrid nanorobots (bioNR), designed to carry drug loaded NP formulations. Table 1 summarizes the advantages and limitations of NP and NR in therapeutic applications.

**Table 1:** Advantages and limitations for therapeutic means of NP and NR.

Advantages	
NP	**NR**
*Enhanced drug solubility*	
A large surface area to volume ratio allows increased drug contact with the solvents [38].	
*Increased drug stability*	
Drugs are encapsulated into porous or holly structures [36].	
*Targeted drug delivery possibility*	
Functionalized NP bind to specific cell receptors [37,45].	Targeted interventions in remote and hard-to-reach areas of the body [39,40].
*Sensing/Diagnostics*	
NP functionalized with bioreceptors can detect biomarkers where fluorescence properties or plasmonic signals of the NP are used for signal read out [46].	Carry bioreceptor molecules and signal transducers that can detect specific biological signals [47].
*Controlled release*	
Can be programmed to release drugs in a controlled manner, allowing sustained and prolonged therapeutic effects [48].	Exhibit significant potential for controlled cargo delivery and release in biological fluids [49].
*Active/Passive*	
Passive structures that rely on external forces to move or interact with their environment [50].	Possessing an active power system that allows them to move and perform tasks [44].
*Improved imaging modalities*	
Serve as contrast agents in imaging techniques, allowing for better visualization of disease markers and accurate diagnosis of conditions [51].	Bio-NR can be utilized for *in vivo* fluorescence imaging and magnetic resonance tracking in deep organs, such as the stomach [52].
*Deep tissue penetration*	
Pass biological tight junctions (e.g. brain, corneal epithelium, retina) due to their small size [53,54].	Their small size and active motion show great potential for penetrating deep tissues [55].
*Antibacterial*	
Positively charged NP disrupt bacterial cell membranes, inhibit biofilm formation, generate ROS, induce oxidative stress, and disrupt bacterial cell components [56,57].	Micro-NR actively deliver cargo and enhance the penetration of antibacterial agents through mechanical force [58].

**Disadvantages**	

*Synthetic efforts*	
May require additional modifications due to limited solubility for some drugs [59].	Require surface modification to improve biocompatibility [60].
*Aggregation*	
Potential for aggregation, which reduces effective surface area [59].	Risk of aggregation, which can compromise their functionality [61]
*Safety/complexity*	
May induce oxidative stress, inflammation, or immune responses eg: carbon-based NP [62]	Potential cytotoxicity, immunogenic responses, complex navigation in biological systems [60]
*Manufacturing challenges*	
Requires precise synthesis methods to control size, shape, and stability [63]	Extremely complex to design and manufacture at a nanoscale level [60]
*Clearance issues*	
May be difficult for the body to eliminate, leading to long-term accumulation [64]	Could face challenges in biodegradability and clearance from the body [44]

While proof-of-concept studies have been essential in advancing NR technologies, the primary focus for the coming years must be on translating the unique capabilities of NR to patient care and addressing unresolved medical challenges [65]. To date, current efforts were largely carried out by research groups on robotics that are actively involved on the development of microrobots for effective drug delivery in cancer therapy applications [66]. The rapid advancement of 3D/4D printing technologies has enabled the quick fabrication of microrobots of various sizes, shapes, and materials, produced in large quantities [67,68]. Table 2 provides an overview of NR designs for advanced medical applications, highlighting their current developmental stages ranging from experimental prototypes to conceptual designs and specific uses.

**Table 2:** NR: Types and their biomedical applications.

Type	Description	Applications	Ref.
**Under investigation and/or in use**			
Microchips	Tiny implantable devices for monitoring physiological parameters, delivering drugs, or stimulating tissues.	Chronic disease management, real-time monitoring, insulin delivery.	[69]
DNA NR	Constructed from DNA molecules, programmable to release therapeutic agents triggered by specific signals.	Precise drug delivery, gene therapy, and cancer treatment.	[70]
Endorobots	Designed for minimally invasive procedures, equipped with cameras and instruments for internal navigation.	Diagnostics, targeted treatments, microsurgery.	[71]
Magnetically guided NR	Controlled using external magnetic fields to navigate to specific sites for treatment.	Targeted drug delivery, hyperthermia therapy, mechanical disruption of blockages, and cancer treatment.	[72]
Photothermal NR	Designed to absorb light and convert it into heat to destroy targeted cancer cells.	Photothermal therapy for cancer treatment.	[73]
Swarmbots	Collective NR work together to perform complex tasks through coordination and communication.	Clearing blockages, targeting cancer cells, multi-site drug delivery.	[74]
**Currently hypothetical**			
Clottocytes	Artificial platelets designed to control bleeding by rapidly forming clots at injury sites.	Emergency medicine, trauma care, surgery.	[75]
Microbivores	NR function as artificial phagocytes, capable of eliminating pathogens like bacteria and viruses.	Treating infections, combating antibiotic-resistant bacteria, biodefense.	[76]
Pharmacytes	Self-powered, computer-controlled medical NR system designed for digitally precise transport, timing, and targeted delivery of pharmaceutical agents to specific cellular and intracellular destinations within the human body.	Personalized medicine targeted drug delivery.	[77]
Respirocyte	Artificial RBC that can carry and deliver oxygen and carbon dioxide more efficiently than RBC.	Respiratory failure treatment, surgery, athletic performance enhancement	[78]
Neural NR	Engineered to interact with the nervous system, monitoring activity, delivering neuroactive drugs, or repairing damage.	Treating neurological disorders, neural monitoring, spinal cord injury repair.	[79]

Considering the interest of NP and NR as therapeutic means, this review focuses in more detail on the state-of-the-art use of these structures for the treatment of lung diseases. It has to be underlined that NR are a novelty for drug delivery with challenges to be overcome (Table 1). Considering the properties of NR to navigate as bloodborne devices, efficient targeted drug delivery is possible with decreasing side effects. However, NR require expensive development strategies currently being of high complexity. It is also generally harder for drug-loaded NR to travel through blood arteries due to the high blood viscosity; however, intensive work is going on to address this issue. This review delves into the progress of application of nanomedicine in respiratory disorders, such as TB, pneumonia, COPD, asthma, and CF, with a particular emphasis on lung cancer management. It outlines various NP-based drug delivery systems tailored for lung cancer management and investigates recent innovations in the field, including the development of inhalable nanomedicines and the utilization of NP-modified microrobotic systems.

One of the current limitations of NR is their reliance on external sources for actuation, which can impede their ability to effectively target and penetrate tumors. They may also struggle to navigate through complex environments, such as biological tissues, without external guidance, significantly impacting their therapeutic efficacy and potential clinical applications [60,80]. To address this limitation, bioNR have recently been developed through bio-hybridizing microorganisms or biological cells with synthetic materials. An ideal bioNR exhibits controlled movement and autonomous drug delivery, reaching difficult-to-access areas of the human body [81,82]. Flagella-carrying bacteria such as *Salmonella-* and *E. coli*-based NR have been of particular interest as they can be easily modified with drug-loaded nanocarriers [82,83]. These bacteria exhibit high motility with some demonstrating tumor-targeting capabilities, making them a promising platform for the targeted delivery of therapeutic agents [84]. Magnetically controlled *E*. *coli* biohybrids have in particular shown great promise. These biohybrids retain their original motility, allowing them to navigate complex biological environments, colonize tumor spheroids, and achieve targeted drug delivery. When tested in 3D collagen gels that mimic *in vivo* tumor environments, these biohybrids demonstrated effective penetration and efficacy [85]. However, the use of bacteria as delivery vehicles presents several limitations, including cytotoxicity, potential immunogenic responses, and short lifespan *in vivo* with the risk of unwanted bacterial proliferation [80]. To mitigate these challenges, several strategies have been explored. Genetic engineering techniques have been used in bacteria to reduce their virulence and enhance their compatibility with host tissue. For example, attenuated strains of *Salmonella* and *E. coli* have been developed by altering bacterial surface proteins or deleting genes responsible for virulence factors, while preserving their motility and tumor-targeting abilities [86]. Additionally, encapsulation techniques, such as the use of biocompatible polymers, have been developed to mitigate the host immune system and to control their release at the tumor site, further minimizing the risk of unwanted immune activation [81].

As an alternative to bacterial-based delivery systems, macrophage-based microrobots have been proposed to deliver drugs or drug-loaded NP through internalization *via* the natural process of phagocytosis or surface conjugation with the cells [80,87]. This approach leverages the natural ability of macrophages to engulf and digest foreign particles [88]. Macrophage-based microrobots are in addition recognized as immune cells, reducing the risk of an immune response against the therapeutic agent with reduced side effects [80,88]. Additionally, macrophages have the ability to migrate, chemotax, and infiltrate tumor cells, and cross the blood barriers of the tumor tissue, altogether enhancing their ability to deliver therapeutic agents directly to the tumor site and interact with tumor cells [89]. Alternatively, unicellular algae *Chlamydomonas reinhardtii* have been shown to propel microscale polymer beads, achieving speeds comparable to unmodified algae, and can control and release these beads using phototaxis and photocleavable linkers. The experiments were performed with polystyrene beads within microfluidic channels and 3D collagen gels to investigate the attachment, motility, and functionality of the biohybrids [90]. Table 3 outlines some examples of bioNR and their application in medical therapies.

**Table 3:** Biohybrid NR: applications in medical therapies.

BioNR	Application	Description	Ref.
**Bacteria-based**			
Chlorella-based microswimmers	Muscle activation and targeted drug therapy	Chlorella microalgae coated with superpara-magnetic Fe_3_O_4_ NP, used for wireless and precise muscle activation and pH-triggered drug release to tumor cells.	[91,92]
Magnetotactic bacteria-based microrobots	Targeted drug delivery	Microrobots utilizing magneto-aerotactic migration behavior of *Magnetococcus marinus* to transport drug-loaded nanoliposomes into the hypoxic regions of tumors.	[93]
***E. coli***-based microrobots	Targeted drug delivery	*E. coli* bacteria attached to drug-loaded polyelectrolyte multilayer microparticles, offering potential for controlled drug delivery.	[82]
***Salmonella***-based microrobots	Cancer therapy	Genetically modified *Salmonella* bacteria designed to deliver therapeutic agents directly to the tumor site.	[94]
**Algae-based**			
*Chlamydomonas*- hybrid microrobots	Antibiotic delivery to the lungs	Antibiotic-loaded neutrophil membrane-coated polymeric NP linked to natural microalgae for active antibiotic delivery in the lungs to treat infections like acute *P. aeruginosa* pneumonia.	[95]
Spirulina -microrobot hybrids	Targeted drug delivery & imaging	Spirulina algae loaded with chemotherapeutic drugs for guided therapy for lung metastasis of breast cancer.	[96]
**Cell-based**			
Sperm-hybrid microrobots	Targeted drug delivery	Sperm cells coated with magnetic NP used for guiding drugs to the female reproductive tract or for targeted cancer therapy.	[97]
Macrophage-based microrobots	Cancer therapy	Macrophage loaded with docetaxel and magnetic NP used to target and infiltrate tumor cells.	[98]

These systems demonstrate varying degrees of effectiveness in targeting specific tissues, controlled drug release, and multifunctionality. Bacteria- and algae-based robots excel in reaching specific areas like hypoxic tumor regions or organs such as lungs, while cell-based systems like macrophage-based microrobots show promise in tumor infiltration. However, significant research gaps remain, including long-term safety assessments, scalability for clinical use, comparative efficacy studies, and strategies to mitigate potential immune responses. Additionally, exploring combination therapies and establishing clear regulatory pathways will be crucial for advancing these innovative technologies towards clinical applications.

## 3 The lung: structure, pathologies, and drug delivery routes

### 3.1 Structure and major pathologies

**Fig. 1:**
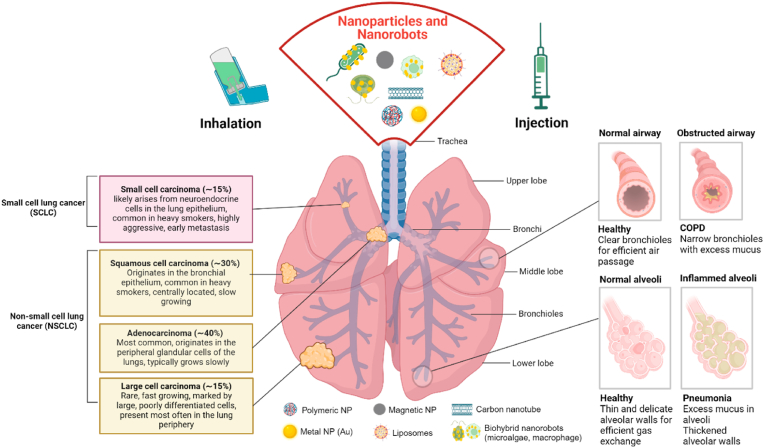
**Overview of lung anatomy, common lung pathologies, and potential drug delivery routes.** Overview of the lung's anatomical structure, highlighting key components such as the airways, alveoli, and lobes. Illustration of various lung pathologies, including different types of lung cancers, their incidences, and characteristics (left), as well as other common lung diseases such as COPD and pneumonia (right). Each pathology is depicted alongside the relevant anatomical regions affected. Additionally, the figure presents different types of NP and NR that are potentially used for the treatment of these lung conditions. Treatment possibilities by inhalation or injection of NP or NR (Reprint with permission from Biorender, agreement number TG27WVU6UB).

Lung diseases affect different parts of the lung, an organ with several key structures that facilitate respiration (Fig. 1). Each lung is surrounded by protective membranes called the pleura and is divided into lobes, with three in the right lung and two in the left [99]. This intricate structure allows for the efficient oxygenation of blood and removal of carbon dioxide, essential for sustaining life. The lung is structured into the trachea, which branches into two main bronchi, further dividing into smaller bronchi and bronchioles. This airway network terminates in clusters of tiny air sacs called alveoli, where gas exchange takes place. The microscopic air sacs are crucial for gas exchange, and when affected by disease, can severely impair breathing.

Alveoli make up the majority of lung tissue. Common alveolar diseases include pneumonia, which results in the inflammation of the alveoli, filling them with fluid or pus and severely impairing gas exchange. This leads to symptoms such as difficulty in breathing, cough, fever, and reduced oxygen levels in the blood [100]. Asthma, on the other hand, affects the airways, particularly the bronchi and bronchioles. It causes these airways to narrow due to inflammation, muscle constriction, and excess mucus production, resulting in coughing, chest tightness, wheezing, and shortness of breath [3]. COPD is a progressive condition that affects both the airways and the alveoli. It includes chronic bronchitis, which causes inflammation and narrowing of the bronchi, and emphysema, which destroys the alveolar walls, leading to airflow obstruction and difficulty exhaling fully. This results in persistent shortness of breath, chronic cough, and increased sputum production [8]. On the other hand, lung cancer can develop in all the different parts of the lung, including the bronchi, bronchioles, and alveoli. The growth of cancerous cells disrupts normal lung function and structure, causing symptoms such as a persistent cough, chest pain, hemoptysis (coughing up blood), and shortness of breath. As the cancer progresses, it can invade surrounding tissues and metastasize, leading to a broader range of symptoms [101,102].

### 3.2 NP-based drug delivery routes to the lung

NP-based drug delivery systems must navigate through several barriers to effectively reach the target tissues. The enhanced permeability and retention (EPR) effect facilitates NP accumulation in tumors due to leaky vasculature and poor lymphatic drainage, and is central to modern anticancer strategies that aim to concentrate therapeutic agents in tumors, while minimizing impact on healthy tissues. However, successful treatment also requires overcoming barriers within the tumor microenvironment, such as the dense extracellular matrix (ECM), cancer-associated fibroblasts, and hypoxia [103]. Beyond merely accumulating in the tumor, NP must penetrate these obstacles and should be internalized by tumor cells to perform the therapeutic action. This is a process that can be achieved by surface-targeting ligands that bind to receptors overexpressed on cancer cells [[104], [105], [106]]. Next to passive targeting of the drugs *via* the EPR effect, active targeting involves modification of the NP surface with ligands which can bind to receptors overexpressed on tumor cells. This promotes cellular internalization and enhances the therapeutic effect compared to non-targeted NP [107]. Challenges remain still, including tumor heterogeneity, potential immunogenicity of some ligands, increased complexity and cost of drug development, and the risk of off-target effects. Recent advances in the field focus on multi-ligand targeting strategies and combining active targeting with immunotherapy to further improve treatment outcomes [108].

The route of administration also plays a crucial role for effective treatment. As of now, there are no orally administered nanomedicines for treating lung diseases in the market [109]. Delivery of drugs *via* injection allows for direct introduction of the therapeutic agent into the bloodstream with injectable drug-loaded NP exhibiting enhanced bioavailability compared to oral administration, as they avoid first-pass metabolism [110,111]. Several FDA-approved NP-based drugs, which can be administered by injection, are on the market such as Abraxane® (Albumin-bound Paclitaxel), approved in 2005 and used among others for treating NSCLC [112]. Abraxane is indeed approved for the first-line treatment alongside carboplatin for locally advanced or metastatic cancer, particularly in patients who are not eligible for curative surgery or radiation therapy [113]. Some other injectable nanomedicines are currently under investigation or undergoing clinical trials for lung cancer (Table 4). CRLX101, a polymeric NP formulation of camptothecin, features promising safety, pharmacokinetics and efficacy, leading to its multinational clinical development for cancers [114]. Phase 2 clinical trials for patients with advanced NSCLC who have failed one or two previous chemotherapy regimens have just been completed [115]. Liposomal irinotecan, ONIVYDE®, demonstrated promising anti-tumor activity in NSCLC patients and is currently in phase 3 clinical trial [116].

**Table 4:** Injection-based NP formulations for NSCLC treatment currently in clinical trials.

Drug name	NP	Drug component	Phase	Clinical identifier number
CRLX101	Polymer	Camptothecin	2	NCT01380769
BIND-014	Polymer	Docetaxel	2	NCT01792479
ABI-007	Albumin	Paclitaxel	2	NCT00777246
NC-6004	Polymer	Cisplatin	2	NCT02240238
Abraxane	Albumin	Paclitaxel	2	NCT00729612
ONIVYDE®	Liposome	Irinotecan	3	NCT03088813
Liposomal Irinotecan	Liposome	Irinotecan combined with cisplatin or carboplatin	4	NCT06467786
LIPUSU	Liposome	Paclitaxel	4	NCT02996214

However, the clinical translation of injection-based formulations can face several challenges. The intravenously administered NP tend to accumulate in the spleen and lymph nodes alongside the liver, as a result of opsonization and sequestration of tissue-resident macrophages, resulting in an unintended distribution of nanotherapeutics to healthy organs [117]. This can cause potential toxicity, immunogenicity, and limited ability to cross biological barriers [118]. Regulatory hurdles and the need for thorough characterization and safety testing also present significant challenges [119]. More importantly, several poorly water-soluble drugs cannot be delivered by injection easily, with needle injections being associated with drawbacks linked to being invasive, connected with the sensation of pain, and of limited use for people with needle phobia. These treatments remain rather expensive and result in a large amount of medical waste, due to the lack of reusability.

The delivery of drug-loaded NP to the lung *via* inhalation has emerged as an alternative to needle-based injections [120]. The reduced thickness of the alveolar membrane, the high alveolar surface area available for drug absorption, and the intense vascularization of the alveoli make pulmonary delivery an excellent approach for drug administration [121]. Following deposition in lung fluids, soluble NP can enter the bloodstream, whereas insoluble NP are cleared by cilia in the airways or by macrophages in the alveoli. The small size of NP enables them to navigate the intricate network of airways and reach deep into the alveoli. Localized drug delivery *via* inhalation to the lungs shows thus promise in treating respiratory disorders including CF, COPD, asthma, TB, and lung cancer while reducing systemic toxicity with rapid onset of action [120,122]. Delivery platforms, such as nebulizers, pressurized metered-dose inhalers, and dry powder inhalers, facilitate the administration of these NP [123]. NP can indeed withstand high nebulization forces and handheld nebulizers were developed from 1930 onwards to help patients with asthma to get epinephrine into their airways [124,125]. Nebulizers can produce aerosol droplets with a consistent size range of 3–6 μm diameter, which facilitates the delivery of suspended NP to the distal lung end [126]. Many inhalation formulations are now approved and these can be categorized into aerosol, inhalation powder, inhalation spray, inhalation liquid, and transformable vapor formulations. However, the airway's defence mechanisms have evolved to prevent external substances from entering the lungs and to clear drugs after they are deposited. Therefore, not all drugs are suitable for inhalation due to factors like molecular weight, solubility, and instability in the lung environment [127]. Polymeric NP, especially those made from biodegradable polymers like poly(lactic-co-glycolic) acid (PLGA) and liposomes are commonly used for inhalable formulations. A study investigated celecoxib-loaded lipid NP (Cxb-NLC) and their lung distribution *via* nebulization in mice. A slower systemic clearance compared to a solution formulation was observed with this formulation [128]. Nintedanib (Nint), an FDA-approved tyrosine kinase inhibitor (TKI) with anti-neoplastic effects in NSCLC, has recently been formulated into inhalable PLGA NP for direct-lung administration [129]. *In vitro* cellular uptake studies showed increased internalization in alveolar epithelial cells and lung fibroblasts, while successfully avoiding uptake by macrophages [129]. Inhalable pirfenidone-loaded liposomes have also been reported as a promising approach for the treatment of NSCLC, as they facilitate enhanced direct drug delivery to lung tissues while reducing systemic side effects [130]. A phase I study on aerosolized sustained-release liposomal cisplatin (SLIT cisplatin) for lung carcinoma patients revealed that it was well tolerated with no dose-limiting toxicity [131]. A phase Ib/IIa study investigating the use of SLIT cisplatin inhalation for treating patients with relapsed or progressive osteosarcoma metastatic to the lung has been completed. ClinicalTrials.gov ID NCT00102531.

While these last examples infer the interest in inhaling NP, attention has to be given to the particle size, as the deposition of NP in the different parts of the lung *via* inhalation is a function of aerodynamics [132]; particles that are too large or too small may not reach the desired lung regions, particularly the lower airways or alveoli and the drug must be soluble in the carrier used for aerosolization [133]. Table 5 summarizes the physico-chemical characteristics of NP which influence the uptake into the lung *via* inhalation. NP often face challenges with loss during exhalation, prompting strategies like incorporating them into larger particles to enhance retention in the lungs [134]. Carvalho et al. conducted a comprehensive review detailing how particle size affects regional lung deposition, summarizing the deposition mechanisms as inertial impaction, gravity sedimentation, and diffusion [135]. In this work, it was outlined that particles with >5 μm diameter deposit generally in the upper respiratory tract, while smaller particles (2–3 μm) target the bronchioles, achieving deep lung deposition. Ultrafine particles (<1 μm) can penetrate the alveolar area, while particles with a diameter <10 nm tend to deposit in the trachea-bronchial area.

**Table 5:** Essential physico-chemical characteristics of particles/NP for uptake into the lung *via* inhalation.

Region	Size [μm]	Surface charge	Characteristics
Upper airways	6-10 [135]	Neutral-slightly positive [136]	Mucoadhesive [137]
Trachea	3-6 [138]	Neutral-slightly positive [136]	Mucoadhesive [137]
Bronchioles	2-3 [135]	Neutral-slightly negative [139]	Tagged with ligands for cell surface receptors [140]
Alveoli	<1 [135]	Neutral-slightly negative [139]	PEGylation [37]
Mucus layer	<0.5 [141]	Neutral-slightly positive [142]	Mucus penetrating coatings, PEGylated [142]

**Fig. 2:**
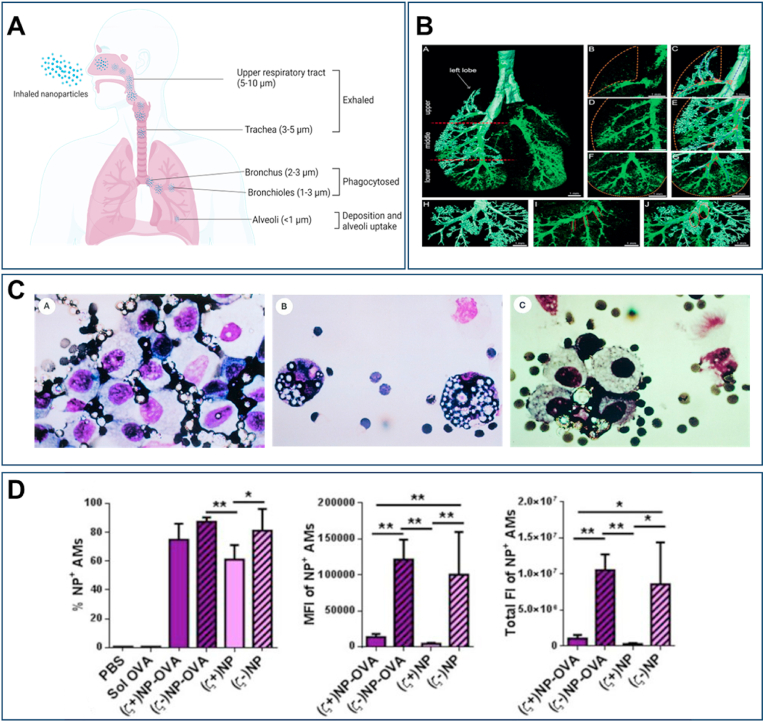
**Distribution of inhaled particles across different lung regions and the influence of particles/NP characteristics on lung deposition**. (**A**) Schematic representation of size-dependent regional deposition of inhaled particles. Reprint with permission from Biorender, agreement number MF27IBJEX1. (**B**) Spatial distribution of inhaled NP. Fluorescence microscopy images showing the distribution of inhaled CL-MOFs-A488. The blue dashed lines indicate the orientation of the trachea and bronchi, while the yellow/orange dashed line marks the lung periphery. The red line shows the angles between airways. The dashed red lines outline the bronchi borders, and the red dashed circles highlight the bifurcations. Scale bar 1 mm. Reproduced with permission from Ref. [145] under Creative Commons Attribution 4.0 International License: https://creativecommons.org/licenses/by/4.0/. Copyright from 2021, John Wiley & Sons. (**C**) Drug delivery using large, porous inhaled particles. Fluorescence images of pulmonary macrophages (large circular objects with dark nuclei) extracted from lungs. **a**: immediately after inhalation of small-nonporous particles; **b**: 48 h post-inhalation of small-nonporous particles; **c**: 48 h post-inhalation of large-porous particles. Inhaled particles appear as white crystals in the images and mostly surround macrophages in **a**, are mainly engulfed within two macrophages in **b**, and remain outside several macrophages in **c**. Reproduced with permission from Ref. [146]. Copyright from 1998, American Physiological Society**.** (**D**) Association of anionic NP with AM from whole lungs of mice. Graphs show from left to right, the percent of positive cells, Median fluorescence intensity (MFI) of positive cells, and total fluorescence of that cell type. Sol OVA: Oval albumin solution, (ζ-)NP: anionic NP, (ζ+)NP: Cationic NP. Reproduced with permission from Ref. [139]. Copyright from 2015, Elsevier. (For interpretation of the references to colour in this figure legend, the reader is referred to the Web version of this article.)

The residence time of particles in the lungs is in addition essential for therapeutic needs and depends on several factors. On average, particles can remain in the lungs for several hours to days. Approximately 90 % of inhaled particles with a diameter greater than 6 μm are cleared within 24 h from the lungs [143]. Lung clearance mechanisms primarily involve mucociliary clearance for particles larger than 5 μm, and macrophage phagocytosis for particles in the 1.5–3 μm range [144]. Alveolar macrophages (AM) can engulf small particles that reach the alveolar region, though their effectiveness varies with particle size. Optimal phagocytosis occurs with particles sized between 1.5 and 3 μm, while particles <1 μm or >5 μm are less prone to phagocytosis (Fig. 2A) [143]. In the case of TB, with AM being the target cells, enhancing macrophage-specific uptake is a desirable characteristic. Smaller particles (<1 μm) generally have a longer residence time in the alveoli, owing to reduced mucociliary clearance and deeper penetration into lung tissue [118]. The distribution of γ-cyclodextrin metal-organic frameworks nanostructures tagged with Alex Fluorescence 488 (CL-MOFs-A488) in the lungs after inhalation was recently demonstrated using fluorescence microscopy (Fig. 2B). This study utilized micro-optical sectioning tomography to achieve high-resolution, three-dimensional visualization of lung anatomy and particle distribution. By co-localizing lung cyto-architectures with fluorescent particles, region-specific deposition patterns were unveiled for the first time with high precision. This approach allowed detailed visualization of the lung's anatomical features, including trachea, bronchioles, and alveoli, and revealed precise spatial distribution patterns of inhaled particles at the single-particle level [145]. Large porous particles (LPP), despite their geometric size of approximately 10 μm in diameter, can mimic smaller particle behavior. These structures exhibit aerodynamic characteristics similar to smaller particles (<5 μm) due to their low density [127]. Their highly porous structure, with densities around 0.1 g/cm^3^ and surface areas exceeding 700 m^2^/g, allows them in addition to effectively evade macrophage recognition and prevent traditional phagocytic mechanisms from efficiently engulfing the particles, thereby enabling prolonged circulation and improved drug release for longer periods of time (Fig. 2C) [146]. It has been demonstrated that inhalable LPP loaded with metformin and docosahexaenoic acid effectively target premetastatic niches in the lungs, reducing tumor metastasis by inhibiting circulating tumor cells (CTC)-endothelial cell adhesion and vascular permeability [147]. Next to size and residence time, surface charge plays a crucial role, with neutral to slightly positive charges improving adherence and retention without triggering excessive immune responses. Anionic particles are more readily phagocytosed by AM compared to cationic particles which primarily clear foreign materials rather than driving adaptive immune responses. In contrast, cationic particles tend to associate more with lung dendritic cells (DCs), initiating adaptive immune responses and are less involved in clearance (Fig. 2D) [139].

Challenges are still encountered in inhaling NP-loaded with drugs. It has to be underlined that the inhalation device used plays an important role in the penetration efficiency of NP, determining whether they can reach deeply into lung tissues and the alveolar area. Some drugs may degrade or lose efficacy when exposed to air or during the nebulization process. Variability in drug delivery, due to factors like breathing pattern and lung anatomy, can lead to less predictable dosing compared to systemic administration. Inhaled drugs can also cause local side effects, such as respiratory irritation, and their effectiveness depends on the proper functioning of inhalation devices [148]. Despite these challenges, pulmonary drug delivery remains a promising approach for treating various lung disorders, with ongoing research focused on overcoming these limitations and expanding its applications.

### 3.3 NR-based drug delivery routes to the lung

Next to numerous NP formulations, NR are currently under investigation as an alternative strategy for the targeted delivery of drugs to the lung [49]. Typically, NR are introduced into the body either through injection into the bloodstream or oral administration. Once in the body, their movement and targeting can be controlled through various mechanisms [44]. The choice of delivery method depends on the NR design, the target site, and the intended application. For instance, magnetically guided NR can be steered through blood vessels to reach tumor sites, while chemically or self-propelled NR might navigate through the gastrointestinal tract [149]. NR propelled by external fields are particularly promising for clinical applications, owing to their fast response time, high biocompatibility, and the ability to be controlled wirelessly and reversibly. Light-driven NR, when exposed to near-infrared (NIR) light irradiation, exhibited enhanced directed-motion behavior which increased their adherence to A549 cancer cells [150].

**Fig. 3:**
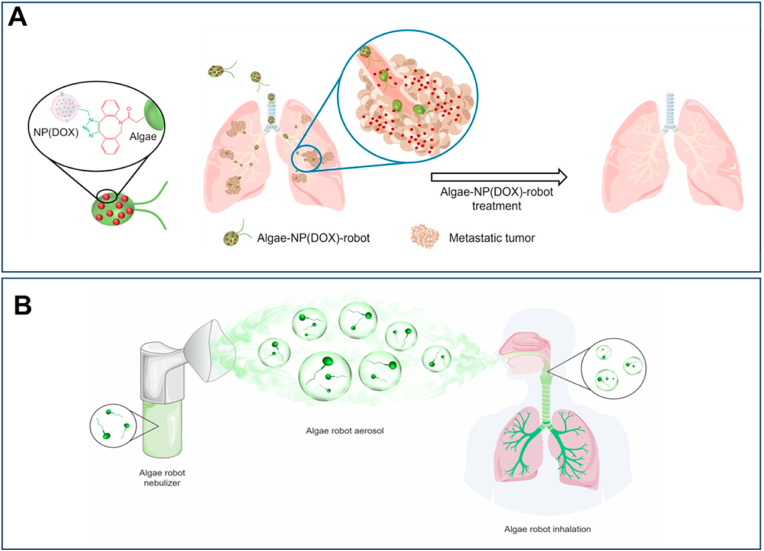
Administration of NR by injection or inhalation for the management of lung diseases (**A**) Intravenous injection of bioNR (algae-NP-DOX-robot) combining green microalgae *Chlamydomonas reinhardtii* with RBC membrane–coated NP containing Dox to treat lung metastasis. Reproduced with permission from Ref. [151] under Creative Commons Attribution 4.0 International License: https://creativecommons.org/licenses/by/4.0/. Copyright from 2024, The American Association for the Advancement of Science. **(B)** Aerosolized *M. pusilla* green algae-based bioNR carrying polymeric NP loaded with vancomycin actively distributed in the lung after inhalation. Reproduced with permission from Ref. [152] under Creative Commons Attribution 4.0 International License: https://creativecommons.org/licenses/by/4.0/. Copyright from 2025, Nature. (For interpretation of the references to colour in this figure legend, the reader is referred to the Web version of this article.)

In recent years, special attention has been given to biohybrid microrobots, which combine living microorganisms like algae or bacteria with synthetic components for improved functionality. Particularly, algae-based bioNR have emerged as a promising strategy for treating lung diseases (Fig. 3). These bioNR leverage the autonomous movement of algae, together with prolonged retention, controlled drug release, and effective distribution throughout the lungs, leading to improved therapeutic outcomes. Fig. 3 highlights the use of NR for targeted delivery *via* injection and inhalation including a hybrid system combining green microalgae *Chlamydomonas reinhardtii* with RBC-coated NP encapsulating doxorubicin (Dox) [151] and *Micromonas pusilla* with platelet membrane-coated polymeric NP loaded with vancomycin, respectively [152]. The Algae-NP-DOX-robot demonstrated substantial improvement in reducing metastatic burden when administered *via* intratracheal injection [151]. Recently, a study by Li et al. presented a novel non-invasive approach for delivering bioNR to the lungs *via* inhalation. By using a nebulizer, *M. pusilla* robots, functionalized with platelet membrane-coated polymeric NP loaded with vancomycin encapsulated in aerosol particles, enabled efficient deposition in the lower respiratory tract. This inhalable system, which eliminates the need for invasive intratracheal administration or anesthesia, offers significant translational potential for clinical applications [152]. Notably, both systems demonstrated significant efficacy in treating lung conditions, including pneumonia and melanoma lung metastasis, by improving drug accumulation, resulting in enhanced survival rates in preclinical models. So, where do we stand in using NP and NR in the important area of treating lung diseases?

## 4 Lung cancer

Lung cancer, characterized by poor prognosis and low overall survival rates, due to late detection and limited therapeutic options, is one of the leading causes of cancer-related deaths globally [153,154]. Lung malignancy, originating from the epithelial cells that line the respiratory tract, is mainly categorized into two major types: NSCLC, which accounts for up to 75 % of lung cancers, and small cell lung cancer (SCLC). Each type exhibits unique biological behaviors, treatment responses, and prognoses [155]. SCLC represents about 10–15 % of lung cancers that are characterized by rapid growth and early metastasis [156]. Emerging technologies, such as liquid biopsies, which analyze circulating tumor DNA (ctDNA) in the blood, offer the potential for earlier precise detection of lung cancer, which would increase the success rate of follow-up therapy [157]. Additionally, advances in next-generation sequencing (NGS) and other genomic profiling techniques are enhancing our understanding of the genetic landscape of lung cancer, paving the way for the more targeted development of novel therapies [158].

Due to the complex pathogenesis of lung cancer, involving multiple signaling pathways, and the need for high drug dosages due to drug solubility problems, treatments are often not effective, making the search for alternative technologies of prime importance [29]. Treatment of lung cancer usually starts with surgery, followed by radio/chemo/immunotherapy. Targeted therapy for NSCLC is largely based on the use of TKI, with a significant number of lung cancer drugs being lately approved by the FDA targeting gene mutations, including EGFR, anaplastic lymphoma kinase (ALK), ROS proto-oncogene 1 Receptor Tyrosine Kinase (ROS1), B-Raf proto-oncogene Serine/Threonine Kinase (BRAF), KRAS Proto-Oncogene, GTPase (KRAS), Neurotrophic Tyrosine Receptor Kinase (NTRK), MET Proto-Oncogene, Receptor Tyrosine Kinase (MET), and Ret Proto-Oncogene (RET) [159]. Despite the emergence of new therapeutic options for patients with advanced NSCLC, significant challenges remain, as most patients experience resistance development and disease progression.

The use of different NP types for targeting lung cancer receptors, such as EGFR, integrins, folate receptors, or CD44, often overexpressed on malignant cells, has found widespread interest [137,160]. PLGA NP, for example, demonstrated cytocompatibility with A549 lung epithelial cells up to 5 mg mL^−1^ [161] and were able to overcome the natural barriers of the respiratory system, including mucociliary clearance and AM phagocytosis, owing to their size being <200 nm [162]. Histological analysis of lung tissue sections, after intratracheal administration of PLGA NP, confirmed that these particles do not induce lung tissue damage and are rapidly absorbed into the bloodstream through type 1 alveolar cells within about 30 min and resist uptake by macrophages [163]. Internalization studies of DNA-loaded PLGA–PEI NP in the Calu-3 human airway epithelial cell line validated the presence of DNA in the endo-lysosomal compartment 6 h post-application [164]. Treatment of NSCLC by inhaling PLGA NP loaded with afatinib revealed decreased side effects when compared to delivery of afatinib orally with 85 % of the afatinib dosage reaching deeper lung areas [165]. Similar findings were observed for inhalable sorafenib-loaded cationically-modified polymeric NP when used to treat NSCLC [166]. PLGA NP coated with a polyethylene glycol (PEG) shell along with pH and reduction responsive disulfide and phenylboronate ester bonds, and an encapsulated platinum complex of curcumin showed improved anti-metastatic activity, offering a promising new strategy for NSCLC therapy [167]. Table 6 illustrates notable research on different NP-based delivery systems for lung cancer management, ranging from polymeric and liposomal to metallic and DNA-based NR. These systems demonstrate significant advancements in targeted drug delivery, enhanced therapeutic efficacy, and reduced side effects. Key features include versatile targeting mechanisms, multifunctional capabilities combining therapies, and innovative approaches to overcome drug resistance. Notably, emerging technologies like DNA NR and macrophage-loaded NP offer promising new strategies. However, research gaps persist, particularly in long-term safety assessments and clinical translation.

**Table 6:** NP and NR-based delivery systems for lung cancer management.

NP	Example	Properties	Application	Ref
Polymer	Poly(β-amino esters) (PBAEs)-based NP encapsulating anti-VEGF mRNA	Encapsulated with synthetic mRNA encoding bevacizumab, an anti-VEGF antibody, optimized for selective lung transfection, inhibits tumor proliferation.	Targeted delivery to lung endothelial cells for NSCLC treatment, anti-angiogenesis, lung cancer gene therapy.	[168]
	Resveratrol loaded poly(glycerol adipate-co-ω-pentadecalactone)	High encapsulation efficiency and cytocompatibility, enhanced potency compared to free resveratrol, reduces cancer cell viability and induces apoptosis.	Enhanced anticancer potency of resveratrol for lung cancer treatment.	[169]
	Arginylglycylaspartic acid (RGD) decorated PLGA NP loaded with cisplatin	PLGA NP with RGD receptor targeting with high drug encapsulation efficiency and controlled drug release for up to 72 h, high biocompatibility, low systemic toxicity, and minimal lung tissue damage, higher cytotoxicity compared to marketed cisplatin injections, with superior pharmacokinetics.	Controlled and targeted co-delivery of cisplatin and upconversion NP (UPNP) in lung cancer therapy.	[170]
Liposomes	RGD peptide-conjugated Dox-loaded liposomes	Target the integrin α(v)β(3) receptor overexpressed in lung cancer and inhibit tumor growth in mice.	Enhanced therapeutic efficacy and reduced off-target effects in lung cancer treatment.	[171]
	Curcumin-loaded liposomes	Enhance uptake and cytotoxicity on A549 lung cancer cells compared to free curcumin, anti-oxidative, anti-inflammatory, and pro-apoptotic.	Inhalation treatment for lung cancer.	[172]
	Peptidomimetic conjugate (SA-5)-conjugated Dox-loaded liposomes	Target EGFR-2 (HER2) overexpressed NSCLC and deliver Dox. Selective cellular uptake and sustained drug release, antiproliferative.	Increased therapeutic efficiency and targeted delivery of Dox in HER2 overexpressing NSCLC.	[173]
	Cisplatin and phenethyl isothiocyanate loaded liposomes	Enhance the toxicity of the drug combination towards NSCLC cells, showing improved efficacy compared to free drugs.	Treatment for NSCLC, offering a more effective approach than administering the drugs separately.	[174]
Lipid	DTX-loaded lipid-based nanoemulsions	Improve bioavailability of the drug, highly selective to carcinoma cells over normal cells, exhibit high colloidal stability, slow drug release, and excellent thermal stability.	Pulmonary delivery system for DTX to target lung cancer.	[175]
	Transferin conjugated afatinib loaded lipid-polymer hybrid NP	Redox-responsive with enhanced drug release in the presence of glutathione, selectively deliver drugs to lung cancer cells and reduce tumor volume.	Platform for NSCLC therapy with reduced systemic toxicity.	[176]
Magnetic	SPION coated with silica layers	SPION coated with silica layers (non-porous, mesoporous, or combined), decreased iron release and enhanced surface properties, and good compatibility with lung cancer cells.	Drug delivery carriers and hyperthermia agents for lung cancer treatment.	[177]
	Mn–Zn ferrite magnetic NP (MZF)	NP-loaded block copolymer micellar system, target A549 lung adenocarcinoma cells and enhance hyperthermia.	Synergistic therapy with radiotherapy, effective cancer hyperthermia treatment	[178]
Metal-based	Gold NP (Au NP) modified with the RGD peptide and loaded with microRNA-320a.	Specific and efficient delivery of miR-320a into lung cancer cells, inhibit proliferation and metastasis, and enhance apoptosis in lung cancer, inhibit Sp1 transcription factor expression.	Integrin αvβ3-targeted therapy, photosensitive therapy by laser irradiation, and gene-targeted therapy with miRNA delivery in lung cancer.	[179]
	Cisplatin-loaded human serum albumin (HSA)-based gold nanoshells (HCP@GNSs)	HCP@GNSs served as drug nanocarriers and mediators for photothermal therapy, showing enhanced cytotoxicity with NIR exposure and improved tumor clearance with no adverse effects, induced the recruitment of dendritic cells, B-cells, and natural killer T-cells in distal tumors to inhibit the growth of the tumors.	Platform for curative lung cancer treatment by inducing immune response inhibiting tumor growth, photothermal therapy for lung tumors.	[180]
	Silver NP (Ag NP) synthesized using *Dicoma anomala* MeOH root extract	Dose-dependent decrease in A549 cell proliferation and significant increase in cell death.	Green nanotechnology-based anticancer treatment for lung cancer, used alone or in combination with PPBa-mediated photodynamic therapy.	[181]
Carbon-based	Functionalized graphene oxide (GO) NP encapsulating quercetin and lurbinectedin	Transparent, smooth surface for drug loading, induce cytotoxic effects and apoptotic cell death by impacting gene expression related to apoptosis (p53, Bax, Caspase-3, and Bcl-2).	Anticancer treatment for lung cancer, specifically targeting A549 and PC9 cell lines.	[182]
	MWCNT conjugated with transferrin and loaded with DTX	Improved aqueous dispersity and biocompatibility with TPGS, higher cellular uptake and enhanced efficacy and safety compared to commercial DTX injections.	Effective lung cancer treatment with reduced oxidative stress.	[183]
	ZnO quantum dots (QDs) on nitrogen and sulfur co-doped porous carbon nanosheets (CNS)	Size-dependent ZnO QDs, fine dispersion on CNS, lower limit of detection of 800 ppb, response to toluene (31.4) with response/recovery times of 18 s and 58 s	Breath analysis for early lung cancer diagnosis, chemi-resistive gas sensing of lung cancer biomarkers (toluene, isoprene, acetone, benzene).	[184]
NR/bioNR	Magnetic tri-bead microrobots	Magnetically guided, attached with NIR photothermal-responsive azobenzene molecules, drug release triggered by NIR irradiation, cytocompatible even at concentrations up to 200 μg mL^-1^, reducing lung cancer cell viability.	Targeted chemo-photothermal therapy for lung cancer.	[185]
	DNA NR loaded with thrombin	Constructed using DNA origami, functionalized with a nucleolin-targeting aptamer, triggers mechanical opening, expose thrombin to induce coagulation at the tumor site, safe and immunologically inert in animal studies.	Targets tumor-associated blood vessels, induction of thrombosis, tumor necrosis, and inhibition of tumor growth, precise drug delivery in cancer therapy.	[186]
	Tetrahedral DNA nanostructures loaded with paclitaxel	Loaded with paclitaxel, effective against paclitaxel-resistant NSCLC, downregulates the expression genes involved in drug resistance genes such as mdr 1 and P-glycoprotein, cytotoxic against NSCLC, pro-apoptotic.	Treatment of paclitaxel-resistant NSCLC, overcome drug resistance.	[187]
	Macrophage-loaded PLGA NP with a divalent metal ion-phenolic network surface modification and Dox.	MPN surface modification for external attachment to macrophages, minimizing intracellular uptake, <cytocompatibility with minimal impact on cell proliferation, acceptable acute toxicity, with no significant damage to major organs or adverse effects in blood parameters, maintain bioactivity and chemotaxis of carrier cells, induce tumor cell necrosis.	Effective in reducing lung metastasis, enhanced drug release by photothermal effect at the tumor site.	[188]
	Macrophage loaded with magnetic iron oxide NP	Encapsulated in macrophages using an insemination-like formulation strategy, modulates tumor suppression, promote macrophage polarization from M2 to pro-inflammatory M1.	Targeting lung cancer through macrophage-mediated delivery of anticancer agents, boost macrophage polarization in the TME favorable for the treatment.	[189]

**Fig. 4:**
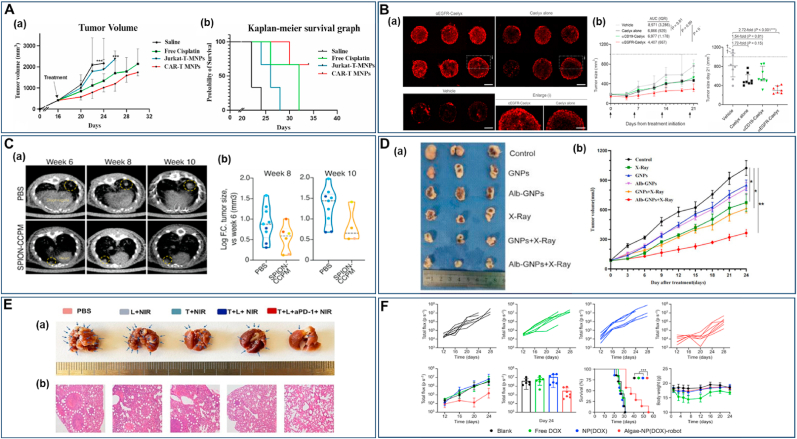
**Recent advancements in lung cancer therapy with different types of NP and NR. (A)***In vivo* assessment of the therapeutic efficacy of CAR-T-MNPs on subcutaneous A549 tumors in nude mice, in comparison to treatments with free cisplatin, Jurkat-T-MNP, and saline, including **(a)** tumor growth delay and **(b)** survival analysis. Reproduced with permission from Ref. [190] under Creative Commons Attribution 4.0 International License: https://creativecommons.org/licenses/by/4.0/. Copyright from 2024, Elsevier. **(B)** Liposomal Dox targets EGFR and suppresses NSCLC. **(a)** αEGFR-Caelyx penetrated and accumulated into the inner region of spheroids *in vitro* and **(b)** significantly suppressed NSCLC progression in a tumor-xenograft model, with tumor size data shown for grouped mice and tumor size on day 21. Scale bar, 50 μm. Reproduced with permission from Ref. [199]. Copyright from 2024, Dove Medical Press Ltd. **(C)** SPION NP reduced lung cancer regrowth after crizotinib treatment. **(a)** Representative μCT lung images from mice treated with PBS and SPION-CCPM at weeks 6, 8, and 10, with yellow circles marking the location of a growing tumor. **(b)** Tumor size at weeks 8 and 10 analyzed by μCT. Reproduced with permission from Ref. [205] under Creative Commons Attribution 4.0 International License: https://creativecommons.org/licenses/by/4.0/. Copyright from 2024, American Chemical Society. **(D)** Albumin-modified gold NP demonstrated enhanced lung cancer radiotherapy. (**a**) Images of tumor tissue under various treatment conditions. (**b**) Tumor growth curves for the different experimental groups. Reproduced with permission from Ref. [208]. Copyright from 2023, Dove Medical Press Ltd. **(E)** CNSI enhanced photothermal-immunotherapy towards cancer metastasis. Mice with metastatic sentinel lymph nodes (SLNs) were randomly assigned to treatment groups as follows: PBS, SLNs (L) + NIR, Tumor (T) + NIR, T + L + NIR, and T + L + NIR combined with aPD-1. **(a)** Imaging of whole lung tissue *ex vivo* with arrows representing metastatic nodules. **(b)** H&E staining of pulmonary tissue showing aggressive metastasis in PBS group and significantly reduced in CNSI- aPD-1 combination. scale bar, 200 μm. Reproduced with permission from Ref. [211]. Copyright from 2024, Elsevier. **(F)** Biohybrid microrobots inhibit the progression of lung metastasis. The therapeutic efficacy of algae-NP(DOX)-robot against melanoma lung metastasis was evaluated by quantifying the bioluminescence intensity of individual mice over time Additionally, total bioluminescence intensity was quantified over time. Reproduced with permission from Ref. [151] under Creative Commons Attribution 4.0 International License: https://creativecommons.org/licenses/by/4.0/Copyright from 2024, The American Association for the Advancement of Science. (For interpretation of the references to colour in this figure legend, the reader is referred to the Web version of this article.)

Fig. 4 illustrates cutting-edge nanomaterial strategies for lung cancer therapy, featuring diverse NP and NR platforms engineered with sophisticated material modifications, each demonstrating enhanced therapeutic targeting, drug delivery, and anti-tumor efficacy through precise material design and functionalization. Chimeric antigen receptor-T-cell membrane-coated PLGA particles (CAR-T-MNP) loaded with cisplatin exhibited equally promising NSCLC treatment outcomes due to sustained drug release over 21 days and increased uptake by A549 cells. *In vivo* studies in a lung cancer nude mice model confirmed that the (CAR)-coated PLGA particles localized to tumor areas and significantly reduced tumor volume growth compared to that of free cisplatin with a better animal survival rate after two weeks post-treatment (Fig. 4A) [190]. T7 peptide-modified NP (T7-CMCS-BAPE, CBT) using carboxymethyl chitosan (CMCS) for co-delivery of docetaxel (DTX) and curcumin displayed a remarkable ability to bind specifically to the transferrin receptor expressed on lung cancer cells. These NP demonstrated dual stimuli-responsive properties, releasing their drug payloads in response to the acidic pH of tumors and elevated reactive oxygen species (ROS) levels [191]. Next to these NP, liposomes and lipidic NP have shown promise in improving the therapeutic efficacy of drugs for lung cancer [192,193], as they can encapsulate both hydrophilic and hydrophobic therapeutic agents, including genes [194,195]. In addition, their enhanced stability with the ability to fuse with cell membranes facilitates the release of therapeutic agents into cells [196]. PEGylated liposomes, such as Doxil® (Dox) and Onivyde® (irinotecan), were one of the first anti-cancer nanostructures to be employed due to their prolonged circulation time and enhanced passive accumulation in solid tumors through EPR effect [197,198]. PEGylated liposomal Dox (Caelyx) targeted to EGFR (αEGFR-Caelyx) was found to accumulate and penetrate the inner region of tumor spheroids with tumor-bearing mice intravenously treated with αEGFR-Caelyx, showing significantly decreased expansion of the tumor (Fig. 4B) [199]. Furthermore, liposomes conjugated with anti-EGFR aptamer-anchored chitosan effectively delivered erlotinib to lung cancer cells with high efficiency [200]. A liposome system coated with hyaluronic acid and chitosan delivered berberine (Ber) and Dox to lung cancer cells efficiently. Ber with its antioxidant properties, counteracted ROS generated by Dox treatment and showed efficient cellular uptake in A549 cells [201].

The targeted delivery of chemotherapeutics to the tracheobronchial region of the lung as an inhaled aerosol was achieved using magnetic NP. A strong magnetic field guided the drug particles to cancerous tissue while overcoming mucociliary clearance, which removes up to 95 % of particles deposited in the tracheobronchial region [202]. Folate-conjugated PEG (FA-PEG) MNP have been developed as efficient imaging agents for active targeting in *in-vivo* models of lung cancers [203]. The combination of magnetic NP with cold atmospheric plasma revealed the promising potential for enhancing lung cancer treatment with a reduction in tumor weight by approximately 60 % compared to untreated controls. This co-treatment led to a marked decrease in EGFR expression and reduced cancer cell proliferation [204]. Recent studies showed that superparamagnetic iron oxide (SPION) NP with core-cross-linked polymer micelles (SPION-CCPMs) effectively target and reprogram tumor-associated macrophages (TAMs) in ALK-positive NSCLC. This reprogramming induced a pro-inflammatory response, enhanced tumoricidal activity, and reshaped the tumor microenvironment from an immunosuppressive to a cytotoxic profile. In a murine lung adenocarcinoma model, microcomputed tomography (μCT) demonstrated that intratracheal instillation of SPION-CCPMs delayed tumor growth compared to the control mice (Fig. 4C). This approach also prevented relapse after initial therapy with crizotinib, suggesting their potential as an adjuvant therapy to improve treatment outcomes [205]. There has always been a significant increase in the use of metal NP such as gold NP (Au NP) for lung cancer treatment. Au NP can be used for gene silencing in lung cancer treatment by delivering antisense DNA or short interfering RNA to silence tumor-related genes [206,207]. Albumin-modified gold NP (Alb-GNP) demonstrated effective accumulation in tumor cells, and their combination with radiotherapy produced a notably enhanced radiosensitizing effect and increased anti-tumor activity favorable for lung cancer decline (Fig. 4D) [208]. Conjugating the anti-EGFR monoclonal antibody nimotuzumab with 27 nm Au NP significantly enhanced therapeutic efficacy against EGFR-overexpressing cancers, as evidenced by lower IC_50_ values compared to free nimotuzumab [209]. The conjugation of silibinin with Au NP led to a 4–5-fold increase in cytotoxicity compared to free silibinin, enhancing its therapeutic efficacy against lung cancer cells due to improved solubility and greater cell penetration ability [210].

Recently, carbon NP suspension injection (CNSI), previously shown to have photothermal effects and approved by China's regulatory authority for clinical use in staining tumor-draining lymph nodes, has been found to effectively eliminate primary tumors when irradiated with near-infrared (NIR) light through localized heating. This process induced immunogenic cell death and enhanced immune responses against distant tumors. Moreover, the damage-associated molecular patterns (DAMPs), produced by the photothermal effect of CNSI, directly activated internal immune cells within sentinel lymph nodes (SLNs), further enhancing the immune response *in vivo*. When combined with anti-PD-1 therapy, CNSI enhanced anti-tumor effects, effectively targeting both metastatic lymph nodes and lung metastases (Fig. 4E) [211]. Significant apoptotic activity was seen for multi-walled carbon nanotubes (MWCNT) stabilized with arabic gum in lung cancer (A549) cells. The investigated MWCNT, which have an average diameter of 10–12 nm and are composed of 99.4 % carbon along with trace elements, increased the expression of key apoptosis-related genes [212]. While carbon nanostructures offer remarkable technological advancements, they come with potential side effects and health risks [213]. Inhalation of MWCNT induced lung inflammation in rats, with varying effects based on the CNT's morphology [214]. Chronic inflammation and immune responses can potentially contribute to long-term health problems, including respiratory and cardiovascular diseases. There is also a risk of bioaccumulation in various organs, which could disrupt normal physiological functions. Some studies even suggest potential carcinogenicity, due to their behavior resembling asbestos fibers [213,215].

A recent study reported *C. reinhardtii-*based bioNR with chitosan-coated iron oxide NP loaded with Dox, featuring magnetic control and light-triggered drug release to deliver drugs to SK-BR-3 cancer cells [216]. In a similar approach, algae-based bioNR were designed to combat melanoma lung metastasis. The algae-NP(DOX)-robot platform was functionalized with Red Blood Cell (RBC) membrane-coated Dox-loaded PLGA NP and maintained the swimming capabilities after drug loading. When administered intratracheally in mice, reduced lung metastatic burden and increased median survival, compared to conventional drug delivery methods, were observed. Further, the microalgae's self-propulsion enabled movement within the lungs of mice, allowing the bioNR to avoid uptake by AM. Monitoring of lung metastasis progression using bioluminescence revealed that these nanostructures suppressed metastasis almost completely after 20 days of treatment. Survival analysis revealed a 40 % increase in median survival to 37 days, with no significant body weight loss, except in the free Dox group, which experienced weight loss, indicating toxicity (Fig. 4F) [151].

**Fig. 5:**
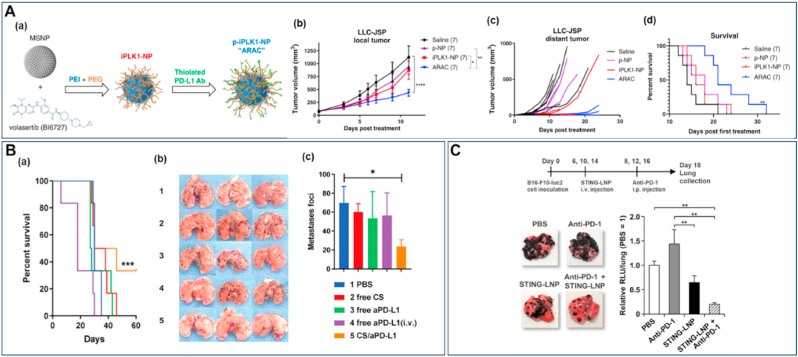
**NP-based strategies for lung cancer immunotherapy. (A)** ARAC targeting PD-L1 and PLK1 for lung cancer management. **a)** Scheme illustrating the synthesis of ARAC. **b)** Local (treated) tumor growth. **c)** Distant (untreated) tumor growth of individual mice. **d)** Kaplan–Meier Survival curve of mice from different treatment groups. Reproduced with permission from Ref. [220] under Creative Commons Attribution 4.0 International License: https://creativecommons.org/licenses/by/4.0/. Copyright from 2022, Springer Nature. **(B)** Inhaled NP-based immunotherapeutics for treating lung metastasis. Results show suppression of lung metastases by inhalation of CS/aPD-L1. **a)** Survival curves of mice subjected to different treatments. **b)** Representative images of lungs collected from mice on day 15 after the indicated treatments. **c)** Number of lung metastatic foci observed on the lung surface following various treatments. Reproduced with permission from Ref. [221]. Copyright from 2021, John Wiley and Sons. (**C)** Combination therapy against anti-PD-1-resistant B16-F10 lung metastasis. The mice received intravenous injections of STING-LNP and intraperitoneal injections of 50 μg of anti-PD-1. The images depict the lungs that were collected for analysis. RLU - relative light unit. Reproduced with permission from Ref. [222] under Creative Commons Attribution 4.0 International License: https://creativecommons.org/licenses/by/4.0/. Copyright from 2021, BMJ Publishing Group.

Nanomedicine in lung cancer immunotherapy is evolving rapidly as another alternative. It is based on triggering anti-tumor immunity by delivering immunotherapeutic agents to tumor microenvironments (TME) and antigen-presenting cells, potentially reducing side effects and increasing the therapeutic efficacy [217,218]. PLK1 inhibition induces cancer cell death but, at the same time, upregulates the programmed cell death protein PD-L1 expression in the surviving cancer cells. Since the PD-1/PD-L1 pathway plays a vital role by which cancer cells escape detection by the immune system, utilizing NP to block these pathways improves the immune system's capacity to identify and target cancer cells [219]. Fig. 5 showcases three innovative NP-based strategies for lung cancer immunotherapy, highlighting the versatility of nanomaterials in enhancing treatment efficacy. Reda et al. have developed injectable NP-based immunotherapy, known as ARAC (Antigen Release Agent and Checkpoint Inhibitor), for co-delivery of a PLK1 inhibitor (iPLK1) called volasertib and a programmed cell death protein 1 (PD-1) antibody using polymer-coated mesoporous silica NP (MSNP) which resulted in an efficient immunotherapy formulation (Fig. 5A). PLK1 inhibition induced cancer cell death but at the same time upregulated PD-L1 expression in the surviving cancer cells, thereby creating an opportunity to target these cells with PD-L1 antibody-conjugated NP. The viability of human A549 cells and metastatic lung tumor model (LLC-JSP) was reduced with the formulation triggered an anti-tumor immune response in a bilateral lung cancer model. Mice with tumors on both flanks that received injections of the ARAC formulation exhibited a significant reduction in the growth of local tumors compared to volasertib-loaded NP without PD-L1 antibody-treated group (iPLK1-NP) or PD-L1 antibody-conjugated NP (p-NP) alone treated group (Fig. 5A). Immune profiling revealed reduced PD-L1 expression and increased CD8^+^ T cell proliferation in tumor-draining lymph nodes as well as a higher population of immune cells and an elevated CD8+/Treg ratio compared to the control group [220]. Inhalable NP, prepared by combining chitosan (CS) with anti-PD-L1 (aPD-L1), for effective pulmonary delivery showed enhanced retention and penetration of aPD-L1 into lung metastases, along with potent immune responses activated *via* the cyclic-di-GMP-AMP synthase (cGAS)-stimulator of interferon genes (STING) pathway. *In vivo* experiments involving mice with melanoma lung metastasis revealed that inhalation of CS/aPD-L1 did not completely eradicate metastatic tumors, but significantly prolonged survival for up to 60 days. On day 15, mice were sacrificed to count metastases on the lung surface, revealing that CS/aPD-L1 treatment led to a notable reduction in the number of lung lesions compared to when given intravenously. This indicates that inhalation of CS/aPD-L1 is an effective strategy for inhibiting lung metastasis and may be a promising approach for treating lung cancers (Fig. 5B) [221]. Lipid NP loaded with STING agonist (STING-LNP) effectively countered resistance to immune checkpoint inhibitors in lung metastases of B16-F10 mouse melanoma, a model known for its resistance to anti-PD-1 therapy. The STING-LNP and anti-PD-1 combination exhibited a synergistic anti-tumor effect specifically in lung metastases (Fig. 5C). STING-LNP activated NK cells, which produced IFN-γ, leading to increased PD-L1 expression in cancer cells and improved anti-PD-1 efficacy. The findings highlighted the potential of STING-LNP to address ICI resistance in treating lung metastases in cancer [222].

Although preclinical findings are encouraging, the complete clinical potential of NP and NR has not yet been achieved. Challenges associated with reproducibility and scalability need to be overcome before a real clinical translation is possible. While most of the NP and NR formulations are investigated for systemic intravenous or oral administration, inhalable NP and NR is part of current promising research with the first clinical trials undergoing. Indeed, inhaled drugs can effectively address issues related to reduced bioavailability at the target site and minimize toxicity associated with the metabolism and excretion of nanomaterials in the mononuclear phagocytic system and kidneys, which are common challenges in oral and systemic administration methods. Besides formulation-orientated advancements, bispecific lung cancer concepts are emerging. For instance, using NP for delivering plasmids encoding chemokines CXCL9, CXCL10, and CXCL11 combined with anti-PD-1 antibody treatment might be interesting alternatives to immunotherapy strategies [223]. In a mouse LLC tumor model, intravenous injection of such NP significantly reduced tumor volume and increased CXCL9, CXCL10, and CXCL11 protein levels in tumor tissues. This treatment also led to a notable increase in CD4^+^, CD8^+^, and CD3^+^ T cell counts. Additionally, NP-treated mice had lower TGF-β levels and higher IL-12 and IFN-γ levels compared to untreated and free pCXCL9/10/11-treated mice, indicating enhanced T cell infiltration and anti-tumor activity [223].

## 5 NP-based strategies for treating respiratory infections

Pulmonary infections are primarily treated *via* oral and intravenous delivery of antibiotics. However, antibiotic delivery *via* oral and intravenous routes is accompanied by a rapid clearance from the circulation. This results in low drug concentrations at the infection site, diminished therapeutic efficacy, and a higher risk of drug resistance [224]. NP are emerging as potential next-generation antibiotic mimetics, due to their inherent antimicrobial activity against both gram-positive and gram-negative bacteria [[225], [226], [227]]. For instance, titanium dioxide (TiO_2_) NP have shown to adhere to the surface of bacterial cells, producing ROS which results in damage of the composition and structure of the cell membrane [228]. Additionally, copper oxide NP (CuO NP) have proven to have significant antibacterial and excellent anti-biofilm activities against *K. pneumoniae*, *S. aureus*, and *A. baumannii* when used at high concentrations of 1000 μg mL^-1^ [229]. It has been demonstrated that the increase in membrane tension, caused by the adsorption of gold NP, leads to mechanical deformation, ultimately resulting in bacterial cell rupture and death [230]. Although their mechanisms of action remain incompletely understood, they include direct bacterial interaction, triggering host immune responses, inhibiting biofilm formation, and disrupting RNA and protein synthesis. Their combination with antimicrobial agents offers a promising approach to tackling the persistent issue of antimicrobial resistance [231].

### 5.1 Pneumonia

Bacterial pneumonia is currently treated with beta-lactams (e.g. amoxicillin, ceftriaxone), macrolides (e.g. azithromycin), fluoroquinolones (e.g. levofloxacin) or tetracyclines (e.g. doxycycline) [232]. Recently, lefamulin and ceftobiprole medocaril sodium (Zevtera) were approved for community-acquired bacterial pneumonia treatment [233,234]. In cases of viral pneumonia, especially those caused by influenza, antiviral medications like oseltamivir or zanamivir may be used [235]. For prevention, vaccines such as CAPVAXIVE, a pneumococcal 21 valent conjugate vaccine approved in 2024, can reduce the risk of certain types of pneumonia in adults [236].

Inhalable NP-based drug delivery systems offer significant benefits for treating lower respiratory tract infections, as they enhance drug interactions with bacteria while minimizing interference with airway barriers [237,238]. For instance, a dry powder inhalation system was introduced using ultrasonic spray freeze drying to deliver liposome-encapsulating ciprofloxacin and colistin (Cipro-Col-Lips) for the treatment of *P. aeruginosa* biofilm-associated lung infections. Effective mucus penetration and biofilm toxin neutralization were demonstrated. In a mouse model, bacterial colonization was reduced by 99.7 %, indicating that this approach represents a promising therapeutic strategy for chronic pulmonary infections [239]. Alternatively, inhalable magnetic NP, formulated as aerosols and directed to specific lung regions with external magnetic fields, enabled targeted antibiotic delivery. When combined with magnetic hyperthermia, these particles have been shown to reduce biofilm and mucus viscosity, thereby enhancing drug and immune cell penetration into the affected areas [240].

**Fig. 6:**
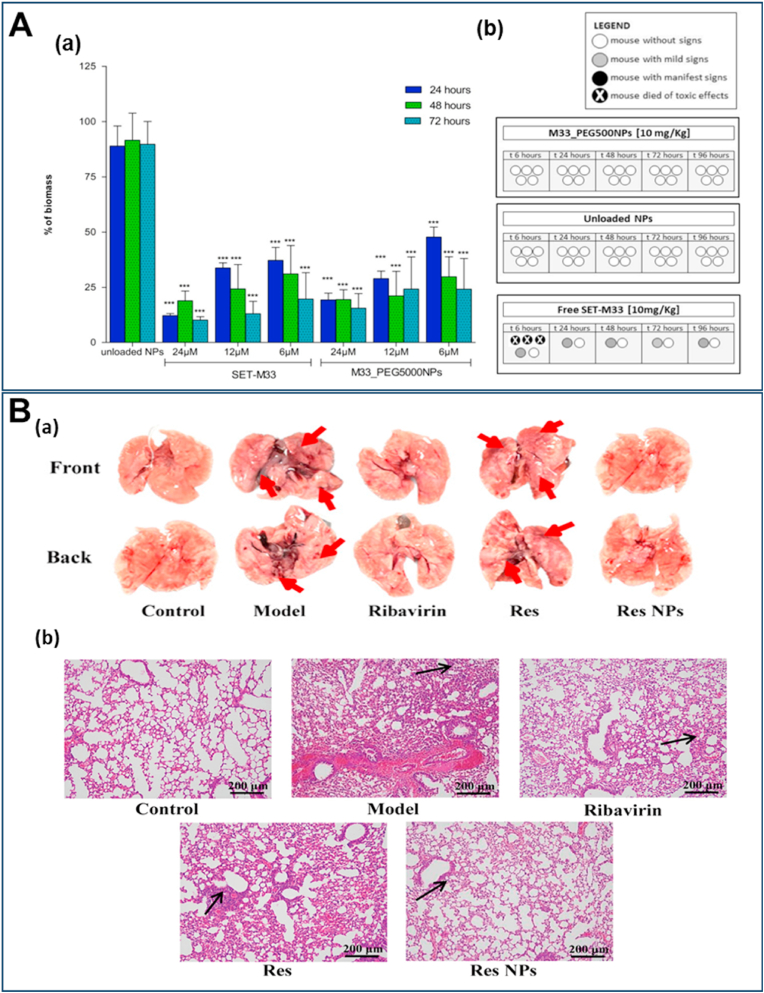
**NP-based strategies to combat bacterial and viral pneumonia.** (**A)** Inhalable polymeric NP for pulmonary delivery of antimicrobial peptide SET-M33. **a**. Percentage of *P. aeruginosa* (ATCC 27853™) biofilm biomass at various incubation times with either free SET-M33 or SET-M33 peptide encapsulated in NP (M33_PEG5000 NP). **b**. The acute toxicity of M33_PEG5000 NP, free SET-M33, and unloaded NP was assessed *in vivo*. Mice (depicted as circles) received an intratracheal inoculation of 10 mg/kg in a single dose and were monitored for 96 h. Various shades of grey and the X symbols within the circles represent the severity of symptoms and mortality observed. Reproduced with permission from Ref. [241] under Creative Commons Attribution 4.0 International License: https://creativecommons.org/licenses/by/4.0/. Copyright from 2023, Multidisciplinary Digital Publishing Institute **(**MDPI). (**B)** Resveratrol NP exhibiting antiviral and anti-inflammatory effects against pneumonia caused by respiratory syncytial virus. (**a**) Mice infected with RSV (model group) displayed pulmonary capillary congestion and edema (indicated by red arrows), compared to the control group. The ribavirin-treated group showed a mostly normal lung tissue appearance. In contrast, the free resveratrol group exhibited mild pulmonary capillary congestion and edema, whereas the resveratrol NP group presented normal lung tissue, characterized by a light pink hue, without any noticeable pulmonary capillary congestion, edema, or hemorrhage. (**b**) Histopathological examination of lung tissue revealed comparable findings, indicating reduced lung pathology in the Res NP treatment group, which exhibited only mild alveolar collapse and inflammatory infiltration (indicated by black arrows), in contrast to the severe lung damage observed in the model group. Scale bar, 200 μm. Reproduced with permission from Ref. [257]. Copyright from 2024, American Chemical Society. (For interpretation of the references to colour in this figure legend, the reader is referred to the Web version of this article.)

Fig. 6 showcases innovative NP-based strategies for combating bacterial and viral pneumonia, highlighting the versatility of NP in respiratory infection treatment. Antimicrobial peptide SET-M33 encapsulated within PLGA NP conjugated with PEG (M33_PEG5000) has been also proposed for targeted ablation of *P. aeruginosa*. Unlike the free peptide, which exhibited toxicity to human bronchial epithelial cells, the encapsulated peptide was biocompatible and displayed antibacterial activity for up to 72 h (Fig. 6A). Furthermore, these NP exhibited superior diffusion across artificial mucus and bacterial biofilm compared to free SET-M33, achieving up to 90 % diffusion across biofilm after 6 h [241]. Inhalable polymeric nanomicelles loaded with azithromycin were employed more recently using the zwitterionic properties of NP to promote the enrichment of the antibiotic-loaded micelles at the site of bacterial infection [242]. Dry powder inhalable formulation of porous PLGA microspheres loaded with heat-resistant, indocyanine green modified phages, have been effective against acute MRSA pneumonia, as they deliver therapeutic phages to deep lung infection sites while avoiding rapid clearance by AM [243]. Inhalable dry-powder formulation, consisting of vancomycin-conjugated iron oxide magnetic NP, encased in a lactose microparticle shell was shown to successfully accumulate vancomycin in the lung tissues of rats and was indicated to have significant potential for localized treatment of MRSA pneumonia. Favorable pulmonary pharmacokinetics were also demonstrated compared to the intravenous administration of the free drug [244]. Inhalable metal-organic-framework NP named ZIF-8 have been developed in addition as sonosensitizers for treating bacterial pneumonia. When exposed to ultrasound, they effectively produce ROS, showing high efficacy in eradicating Gram-negative, multidrug-resistant bacteria *in vitro*. Delivered directly to lung infection sites through aerosolized intratracheal inoculation, these NP enabled targeted sonodynamic therapy, effectively eliminating bacteria in both immunocompetent and immunodeficient mouse models [245]. Recently, Dong et al. developed an inhalable catalase (CAT)-tannic acid nano-assembly (CT@LVX) for targeted delivery of levofloxacin and neutralization of hydrogen peroxide secreted by *S. pneumoniae*. CT@LVX demonstrated significant efficacy in eliminating *S. pneumoniae*, decreasing lung injury, and achieving 100 % survival in pneumonia-affected mice, outperforming traditional antibiotic treatments in a mouse model of clinically isolated *S. pneumoniae*-induced pneumonia [246]. Another notable example was the conjugation of meropenem, a broad-spectrum carbapenem antibiotic, to NP, resulting in the killing of meropenem-resistant *K. pneumoniae* [247]. The combination of levofloxacin (LFX) and lysozyme (LYS) demonstrated enhanced antibiofilm activity, with over 85 % eradication of preformed biofilm at sub-minimum inhibitory concentrations. In a pharmacodynamic study on *S. aureus* infected rats, inhalable LFX-LYS loaded liposomes significantly reduced microbial burden and inflammatory markers in lung tissues and bronchoalveolar lavage fluid (BALF), while showing good safety in acute toxicity assessments [248]. The NP-based formulation is also administered intranasally to enhance local drug delivery and efficacy in treating respiratory infections. For instance, a chitosan-based muramidase Cpl-1 delivery system was administered intranasally, significantly reducing bacterial loads in the lungs, blood, and spleen in a pneumococcal pneumonia model. This approach improved treatment outcomes by lowering infection levels and inflammation while decreasing cytokine levels compared to other treatment groups [249].

Alternatively, injection-based *in vivo* studies have been conducted using NP. ROS-responsive polyurethane NP loaded with the antibiotic azithromycin and ibuprofen efficiently scavenged ROS, eliminated *P. aeruginosa*, and modulated inflammation in both *in vitro* and *in vivo* models. Administration of these nanostructures *via* the tail vein reduced inflammation and epithelial cell apoptosis [250]. NP, formed by the electrostatic self-assembly of hyaluronic acid (HA) and polymyxin B (PMB), have shown a tendency to accumulate in the lungs, targeting CD44 receptors at inflammation sites. This facilitates the release of PMB to engage with bacterial lipopolysaccharides [251]. A NP-based targeted delivery strategy using platelet-derived extracellular vesicles (ECV) to deliver dexamethasone by injection in acute pneumonia has been proposed [252]. Recently, a biocompatible nano-formulation composed of liposomes and dendritic mesoporous silica has been developed to enhance water solubility and reduce the hemolytic activity of the hydrophobic antimicrobial peptide L30. In mice models infected with *S. aureus*, injection of these nanoformulations resulted in improved therapeutic effects, including reduced inflammatory cytokines (TNF-α and IL-1β) levels in plasma and better recovery compared to free L30 by day 14 [253]. Dendritic mesoporous organosilica NP loaded with PMB (PBSi-NP) featured similar effects on carbapenem-resistant *K. pneumoniae*. PBSi-NP effectively scavenged LPS, enhanced bacterial internalization, and released PMB in an acidic environment typical of infection. They exhibited reduced toxicity towards human embryonic kidney cells (HEK 293) and achieved superior targeting of lung cells compared to free PMB, effectively treating pneumonia in a CRKP-induced mouse model and significantly improving survival rates [254]. Lately, neutrophil membrane-coated NP loaded with KLA peptides were able to effectively overcome antibiotic resistance by releasing KLA, reducing intracellular bacteria, and lowering caspase-1 activity, improving survival in a *K. pneumonia* infection model mice with good biocompatibility [255].

Unlike bacterial pneumonia, viral pneumonia can be more challenging to diagnose and treat. Common causes of viral pneumonia include influenza virus, respiratory syncytial virus (RSV), coronaviruses (such as SARS-CoV-2), adenoviruses, and parainfluenza viruses [256]. Respiratory syncytial virus (RSV)-induced pneumonia in children is widespread and poses significant mortality risks, with a pressing need for effective treatments. Resveratrol (Res) has shown antiviral and anti-inflammatory properties, but its poor water solubility limits its use. To overcome this hurdle, resveratrol nanoparticles (Res NP) were developed, enhancing water solubility and dissolution rates. In *in vitro* studies, Res NPs inhibited RSV replication and reduced pro-inflammatory cytokines. Nebulized inhalation administration improved drug retention in the lungs, demonstrating significant efficacy in reducing RSV viral load and improving the pulmonary microenvironment in RSV-infected mice [257] (Fig. 6B). Recently, another study assessed PLGA NP loaded with Chlorine E6, a photosensitizer that generates ROS and luminal to treat viral pneumonia. In murine models of vesicular stomatitis virus-induced pneumonia, these NP reduced inflammation, improved survival rates, and enhanced antiviral protection by promoting apoptosis in infected cells [258]. Table 7 comprises various antimicrobial agent-loaded NP designed for treating respiratory infections, highlighting their mode of delivery.

**Table 7:** Examples of antimicrobial agent-loaded NP for treating respiratory infections.

Antimicrobial agent	Nanostructure	Respiratory infection	Administration route	Ref.
Rifampicin	PLGA/mannitol	TB	Inhalation	[259]
Rifampicin, Isoniazid Pyrazinamide	PLGA	TB	Oral	[260]
Polymyxin B	PLGA-PMB- acid	*P. aeruginosa*	Nebulization	[261]
Clarithromycin	PLGA	*S. aureus* and *M. abscessus* in TB	Aerosol	[262]
Esculentin-1a	PLGA	*P. aeruginosa*	Intratracheal	[263]
Ciprofloxacin	Poly(2-ethyl-2-oxazoline)	Lower respiratory tract infections	Inhalation	[264]
Amikacin	Liposome	Non- TB mycobacterial lung infection	Inhalation	[265]
Licorice extract	Liposome	TB	Inhalation	[266]
Tobramycin	Liposome	*P. aeruginosa*	Intratracheal, Inhalation	[267,268]
Benzothiazinone (BTZ); Levofloxacin (LVX)	Silica NP; Liposomes	TB	Inhalation	[269]
Bedaquiline	Nanoemulsion	TB	Oral	[270]
Colistin	Polymeric NP	*P. aeruginosa*	Inhalation	[271]
SET-M33	Dextran NP	*P. aeruginosa*	Inhalation	[272]
Rifabutin	Solid lipid NP	TB	Inhalation	[273]
Levofloxacin	Solid lipid NP	TB	Inhalation	[274]
F1-V	Polyanhydride NP	*Yersinia pestis* infection in *Pneumonic* plague	Intranasal	[275]
MRSA-specific antibody	Au@Ag core-shell NP	MRSA-related pneumonia	Intravenous injection	[276]
Isoniazid pyrazinamide	Chitosan	TB	Inhalation	[277]
Pneumococcal surface antigen A	Chitosan-DNA NP	*S. pneumoniae*	Intranasal	[278]

### 5.2 Tuberculosis (TB)

The use of inhalable nanostructures has also met success for the delivery of anti-TB drugs to the lungs. Liposomal amikacin has been approved for managing mycobacterial infections [279]. The effects of bedaquiline encapsulated in fucosylated *versus* nonfucosylated liposomes for targeted aerosol delivery to the lungs were explored, with increased liposomal uptake in macrophages observed upon targeting. Although antibiotic release was altered by pulmonary surfactant, high efficacy against *Mycobacterium abscesses* was maintained by dry-powder microparticles of bedaquiline-loaded liposomes, with enhanced bacterial killing potential demonstrated by the fucosylated variants [280]. Inhalable N-acetylcysteine (NAC) loaded PLGA-based mucus-penetrating particles (NAC-PLGA-MPPs) showed promising results for TB treatment, due to their good aerosol performance. *In vitro* assay revealed that NAC-PLGA-MPPs exhibited four times greater antibacterial activity against the MTB H37Rv strain compared to pure NAC and could overcome mucus barriers [281]. Similar findings were reported for linezolid-loaded PLGA NP with sustained drug release for up to 12 h [282].

### 5.3 COVID-19

In the case of COVID-19, lipid NP proved their efficiency in stabilizing mRNA and were one of the great examples of commercialization [283]. Nanoformulated dexamethasone delivered *via* inhalation using lipid NP targeted the interleukin receptor and achieved better management of severe symptoms associated with post-COVID-19 pulmonary fibrosis [284]. Secondary COVID-19 infections were targeted with thymoquinone (TQ) loaded poly(ester amide)-based NP [285]. Hesperidin-loaded chitosan NP (HPD/NP) have been designed for nasal delivery to help manage cytokine storm syndrome (CSS) and acute lung injury (ALI)/acute respiratory distress syndrome (ARDS), including COVID-19-related cases. Studies conducted *in vitro* and *in vivo* demonstrated that HPD/NP significantly enhanced cellular uptake in inflamed environments, leading to notable reductions in lung damage, inflammatory cytokines, and vascular permeability in mouse models, outperforming free hesperidin [286]. In the realm of therapeutics, various NP have been designed to disrupt the binding of SARS-CoV-2 to the angiotensin-converting enzyme 2 (ACE2) receptor, effectively blocking viral entry into host cells. This is crucial for early intervention in infections, as it can halt viral proliferation long enough for the immune system to respond [287]. Orally administered ivermectin-loaded PLGA-based NP enhanced the drug's therapeutic potential by targeting key elements involved in the virus's ability to infect human cells, specifically the viral spike protein and its receptor. By decreasing the expression of these proteins, the NP delivery system could significantly lower transmission rates of SARS-CoV-2. Additionally, the inhibition of nuclear transport activities linked to viral replication suggests a novel mechanism of action that could further hinder the virus's ability to propagate [288]. AM-like NP were engineered for antiviral therapy by coating polymeric cores with membranes derived from AM, providing them the same surface receptors as actual AMs, including the coronavirus receptor and various cytokine receptors. This design allows them to function as AM decoys, blocking coronavirus entry into host cells and absorbing proinflammatory cytokines. With the addition of photothermal inactivation, these NP exhibited both antiviral and anti-inflammatory effects, which are further amplified when subjected to NIR irradiation, enhancing their therapeutic efficacy [289].

COVID-19 can cause severe complications, including acute liver injury (ALI) and acute respiratory distress syndrome (ARDS). ARDS is a common and serious consequence of COVID-19, characterized by intense inflammation, severe damage to the epithelial/endothelial barrier, and alveolar edema [290]. ALI, while less common, has been observed in COVID-19 patients and may result from direct viral infection of liver cells, drug-induced liver injury, or systemic inflammation. Both ARDS and ALI in COVID-19 patients are often linked to cytokine storm syndrome, where an overwhelming inflammatory response leads to multi-organ dysfunction [291,292]. The management of these complications focuses on supportive care and addressing the underlying inflammatory processes, with ongoing research into targeted therapies to improve outcomes. Magnetically controlled NR, named MAGICIAN, created recently by Chen et al. by fusing neutrophil membranes onto iron oxide NP (Fe_3_O_4_ NP) has been developed to address this issue. MAGICIAN showed effective neutralization of inflammatory cytokines like IL-6, TNF-α, and IFN-γ *in vitro* and was magnetically guided to liver sites, improving cytokine clearance. In a mouse model of acute lung injury, MAGICIAN significantly reduced inflammation and demonstrated a favorable safety profile during systemic administration [293]. Alternatively, triangular DNA origami nanostructures modified with R9 peptides (tDONs-R9) were developed as a nebulizable therapy for targeted delivery to the deep alveolar regions, enhancing macrophage uptake. In a mouse model of LPS-induced injury ALI, nebulized tDONs-R9 treatment significantly lowered ROS levels, reduced pro-inflammatory cytokine expression, and decreased neutrophil infiltration, while promoting the polarization of macrophages towards the anti-inflammatory M2 phenotype [294].

Several NP-based formulations have successfully progressed to clinical trials, demonstrating their potential for improving the treatment of respiratory infections. These ongoing trials aim to evaluate their safety, efficacy, and ability to enhance targeted drug delivery while minimizing resistance and side effects. Continued advancements in nanomedicine are expected to pave the way for more effective and accessible respiratory infection treatments. Table 8 summarizes the various NP-based medicines currently in clinical trials for respiratory infections.

**Table 8:** NP-based therapeutics undergoing clinical trials for respiratory infections.

NP-based vaccine/medicine	Disease target	Phase	Aministration route	Clinical identifier number
Amikacin liposome inhalation suspension	Non-TB mycobacterial lung infections	3	Inhalation	NCT04677569
Liposomal lactoferrin	COVID-19	2/3	Oral and intranasal	NCT04475120
Colloidal AgNP	COVID-19	–	Oral and inhalation	NCT04978025
Lipid NP-encapsulated mRNA vaccine	COVID-19	1/2	Intramuscular injection	NCT04566276
Mesenchymal stem cells exosomes	COVID-19	1/2	Inhlation	NCT04491240
T-cell derived exosomes	COVID-19	1	Inhalation	NCT04389385
NP formulation of remdesivir	COVID-19	1	Inhalation	NCT04480333
AgNP	COVID-19	–	Mouth spray and nasal lavage	NCT04894409
Ferritin NP vaccine	COVID-19	1	Intramuscular injection	NCT04784767
Exosome-based therapy	Carbapenem-resistant gram-negative lung infections	1/2	Inhalation	NCT04544215
Liposomal amphotericin B	Invasive pulmonary aspergillosis	1	Inhalation	NCT04267497
Mesenchymal stem cells exosomes	SARS-CoV-2 associated pneumonia	1	Inhalation	NCT04276987
Liposomal ciprofloxacin	*P. aeruginosa* management in non-CF bronchiectasis	2	Inhalation	NCT00889967
Pulmaquin (ciprofloxacin dispersion)	*P. aeruginosa* management in non-CF bronchiectasis	3	Inhalation	NCT02104245
Liposomal Amikacin (ARIKAYCE™)	Bronchiectasis with *P. Aeruginosa* infection	2	Inhalation	NCT00775138

## 6 NR-based strategies to combat respiratory infection

**Fig. 7:**
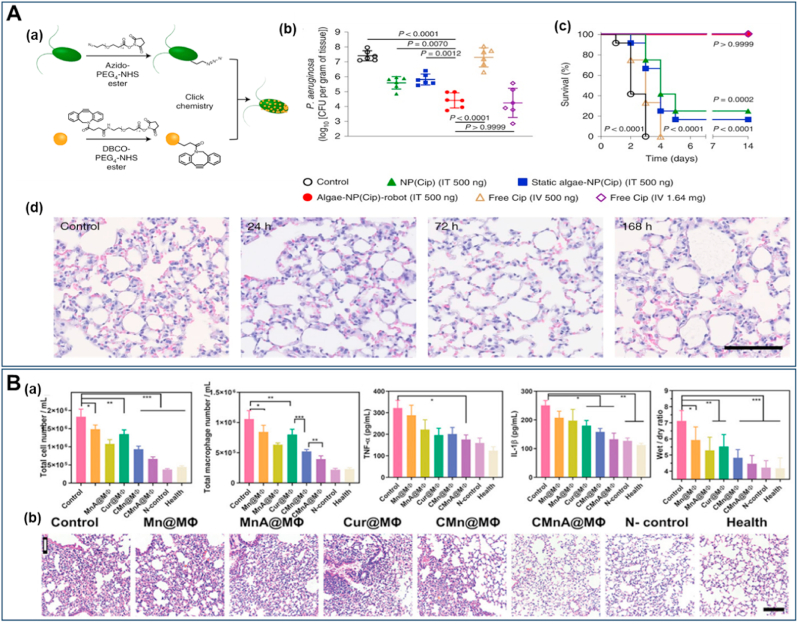
**BioNR for the treatment of pneumonia.** (**A**) NP-modified microalgae robots for *in vivo* antibiotic delivery to treat acute bacterial pneumonia. (**a**) Schematics of the functionalization of *C. reinhardtii* with drug-loaded NP using “click” chemistry approach. To assess the antibacterial effectiveness against *P. aeruginosa* pneumonia, ciprofloxacin (cip)-loaded algae robots (algae-NP(Cip)-robot) and control samples were intratracheally administered 30 min post-bacterial inoculation. The *in vivo* antibacterial efficacy was evaluated for the control TAP medium, NP(Cip), static algae-NP(Cip), and algae-NP(Cip)-robot, all administered at a dosage of 500 ng *via* intratracheal delivery. (**b**) Bacterial enumeration was conducted and (**c**) survival studies were performed. (**d**) Histological examination of lung tissue sections collected at 24, 72, and 168 h post-administration showed minimal leukocyte infiltration, preserved lung tissue architecture, and no indications of inflammation. Scale bar, 100 μm. Reproduced with permission from Ref. [95]. Copyright from 2022, Springer Nature. (**B**) Chemotaxis-guided self-propelled macrophage motor designed for the targeted treatment of acute pneumonia. (**a**) After 24 h of treatment total cell count, total macrophage count and inflammatory cytokine levels in the engineered cell-treated mice, with the most significant reduction observed in the CMnA@MФ-treated group. (**b**) Histological analysis of lung tissue confirmed that CMnA@MФ treatment alleviated pulmonary edema and reduced alveolar injury. Scale bar, 100 μm. Mn@MФ: MnO_2_ NP-engineered macrophages treated acute pneumonia mice, MnA@MФ: intracellular MnO_2_ aggregates-engineered macrophages treated acute pneumonia mice, Cur@MФ: Curcumin-loaded macrophages treated acute pneumonia mice, CMn@MФ: Curcumin@MnO_2_ NP-engineered macrophages treated acute pneumonia mice, CMnA@MФ: intracellular Curcumin@MnO_2_ aggregates-engineered macrophages treated acute pneumonia mice, N-control: negative control, CMnA@MФ-treated healthy mice, healthy: untreated healthy mice. Reproduced with Permission from Ref. [296]. Copyright from 2023, John Wiley and Sons.

While plenty of various nanostructures were investigated for different respiratory infections, where are we standing with NR? NR represent a promising technology for treating respiratory infections, as illustrated in Fig. 7, which highlights bioNR strategies for pneumonia treatment. The seminal work on a motile drug delivery system was based on the use of algae-based bioNR for treating bacterial pneumonia [95]. It featured ciprofloxacin loaded neutrophil membrane-coated polymeric NP linked to natural microalgae using “click” chemistry. These bioNR demonstrated fast movement (>110 μm/s) within simulated lung fluid, enabling deep tissue penetration and minimizing clearance by AM. After intratracheal administration, they remained in the lungs for over two days. In a mouse model of acute *P. aeruginosa* pneumonia, these microrobots notably decreased bacterial load and mortality and exhibited negligible toxicity [95] (Fig. 7A). Recently, in a mouse model of acute MRSA pneumonia, algae bioNR functionalized with platelet membrane-coated NP loaded with vancomycin demonstrated therapeutic efficacy. Using a nebulizer, picoeukaryote algae robots were encapsulated in aerosol particles, enabling efficient deposition in the lower respiratory tract. The microrobots retained motility (∼55 μm s^−1^) post-nebulization, ensuring homogeneous lung distribution and prolonged retention (>5 days) [152].

An antibacterial NR featuring a cell wall-binding domain (CBD)-endolysin sensor, which specifically detects and binds to MRSA cells, and an iron oxide/silica core-shell actuator was lately described [295]. Upon receiving a radiofrequency electromagnetic stimulation signal, the NR induced MRSA cell death through localized heat and ROS, achieving 99.999 % bacterial removal within 20 min. The efficacy of these NR was confirmed in a mouse model of skin infection [295]. In addition, this approach holds promise for treating MRSA-induced lung infections, where the targeted NR could be delivered to the lungs to detect and kill MRSA in respiratory tissues, effectively reducing bacterial load. This makes it a highly specific, resistance-free nanomedical strategy to combat MDR MRSA infections in various organs, including the lungs [295]. Manganese dioxide NP modified with β-cyclodextrin and adamantane were self-assembled into large aggregates within macrophages. Inflammation was reduced by the depletion of H_2_O_2_, and oxygen was generated to propel cell movement, allowing for faster infiltration into inflamed lungs in *in vivo* studies. These self-propelling motorized cells served as active carriers for targeted drug delivery, achieving deep tissue penetration within inflamed lung areas and facilitating localized drug release when injected intravenously in mice. This approach supported synergistic anti-inflammatory effects by promoting M2 macrophage polarization. When loaded with curcumin, the macrophages were guided by chemotaxis, utilizing self-propelled movement to deliver curcumin effectively, resulting in the treatment of acute pneumonia through immunoregulation (Fig. 7B) [296].

Alternatively, RBC have garnered significant attention as promising drug carriers, particularly for improving delivery efficiency to intravascular targets like lungs. RBCs have been employed as carriers in cellular hitchhiking strategies to extend circulation time and increase lung-targeting efficiency of nanoparticulate drug delivery systems, leading to enhanced lung accumulation and reduced uptake by the liver and spleen [297]. In a recent study, a drug delivery system using RBCs to transport liposomes *via* host-guest interactions was developed for treating acute pneumonia. In this approach, β-cyclodextrin-modified RBCs were linked to ferrocene-modified liposomes, allowing the liposomes to hitchhike on RBCs and extend their circulation time in the body. The liposomes were specifically released in the inflamed lungs due to the high levels of ROS in the inflammatory microenvironment. This targeted delivery system effectively reduced lung edema, lowered inflammatory cytokines, and repolarized macrophages in mice with acute pneumonia [298].

The field of NR is currently under expansion to the theranostics area, with for example plasmonic-magnetic NR having been developed to detect COVID-19 utilizing Fe_3_O_4_ core for magnetic propulsion and precise nucleic acid transport. The swarming behavior of the magnetically actuated NR intensified micromixing and enabled active targeting, which in turn improved binding kinetics [299]. Mobile self-propelled NR are also utilized to enhance biosensing systems by enabling real-time identification and isolation of biotargets. In a study, magnetic microrobots were introduced, which utilize collective self-assembly through an immuno-sandwich assay, leading to significantly improved sensitivity and a reduced detection limit of SARS-CoV-2 virus particles by an order of magnitude compared to traditional methods [300]. Additionally, a novel nanoplasmonic biosensor, termed nanoscale robot hand structure (Nano RHB), was introduced for the rapid and specific capture of adenovirus particles. The Nano RHB, enhanced with branched gold nanostructures, achieved ultra-sensitive detection of 100 copies/mL through a one-step sandwich method, with consistency observed in comparison to standard assays and high specificity noted for various viral vectors and pseudoviruses. This platform could be highly beneficial for detecting the COVID-19 virus, due to its rapid, direct, and specific quantification of viral particles [301].

## 7 Advancement of nanotechnology in other common respiratory disorders

### 7.1 Chronic obstructive pulmonary disease (COPD)

COPD, marked by chronic lung inflammation and airflow limitation, can lead to obstructive ventilatory impairment due to emphysema, with current treatments mainly managing symptoms or requiring lung transplantation. Previous findings have indicated that administration of 1.0 mg/kg of the synthetic retinoid Am80 led to the repair of collapsed alveoli and enhancement of respiratory function in a mouse model of elastase-induced emphysema [302]. However, the FDA-calculated clinical dose is estimated at 5.0 mg/60 kg, prompting the need to reduce this amount for powder inhaler formulation. To address this, disulfide (SS)-cleavable proton-activated lipid-like material (SS-OP) which releases the drug by the cleavage of disulfide bonds was developed. Am80-loaded SS-OP lipid NP were efficiently absorbed by cells through ApoE, enabling the release of Am80 into the nucleus *via* RARα [303]. Anti-inflammatory treatments assessed for COPD management encompassed inhaled corticosteroids, oral glucocorticoids, phosphodiesterase inhibitors, antibiotics, and monoclonal antibodies, such as benralizumab and mepolizumab, which target specific inflammatory mediators [304]. When inhaled corticosteroids were used alongside long-acting bronchodilators, they have been particularly effective in decreasing the risk of exacerbations. Recent developments in NP formulations of corticosteroids and beta-agonists have in parallel demonstrated to be ideally adapted to treat COPD due to enhanced lung deposition, prolonged drug release, and improved tissue penetration, leading to more effective management of COPD [305,306]. PLGA NP are in particular well adapted to treat inflammatory diseases as one of their degradation products, lactic acid, has an intrinsic immunosuppressive, and anti-inflammatory effects [307]. Budesonide, an inhaled corticosteroid, and theophylline, an orally administered bronchodilator with a narrow therapeutic range, are for example applied in COPD treatment but pose risks due to theophylline's side effects at high concentrations [308]. Poly-lactic acid (PLA) NP co-encapsulating theophylline and budesonide exhibited sustained drug release over 24 h, with strong lung permeability and minimal cytotoxicity in lung epithelial cells. Nebulization produced high fine particle fractions for both drugs, with 75 % for theophylline and 48 % for budesonide [309].

Lipid-polymer NP have been assessed to address glucocorticoid resistance in COPD. This resistance is linked to the reduced expression of the glucocorticoid receptor (GCR) by senescent CD28nullCD8+ pro-inflammatory lymphocytes found in the peripheral blood of individuals with COPD [310]. Composed of a PLA core encapsulating manganese-centered porphyrin molecules (Mn-porphyrin dimer) as antioxidants and a cationic lipid shell that binds to plasmid DNA encoding histone deacetylase 2 (HDAC2), these nanoplatforms demonstrated the ability to reduce ROS levels, restore HDAC2 expression, enhance steroid sensitivity, and decrease inflammation. This approach also showed potential in reversing mitochondrial dysfunction, marking it a promising therapeutic strategy for COPD [311]. Curcumin loaded mPEG-PLGA NP exhibited lately a greater efficacy in reversing corticosteroid resistance compared to free curcumin [312]. Nasal instillation of multi-shell NP, featuring a calcium phosphate core coated with siRNAs targeting pro-inflammatory mediators encapsulated in PLGA and topped with a final outer layer of polyethylenimine, resulted in a reduction of inflammatory cytokine gene expression and a significant improvement in lung inflammation in mice with TH1 cell-mediated lung inflammation and in an influenza infection model [313]. Lately, inhalable beclomethasone dipropionate dry powders were prepared using a PHEA-g-RhB-g-PLA-g-PEG copolymer, which consists of grafted components, including rhodamine (RhB), PLA, and PEG onto an α,β-poly(N-2-hydroxyethyl)DL-aspartamide (PHEA) backbone. These powders exhibited controlled release, excellent biocompatibility, and enhanced efficacy compared to conventional formulations for the treatment of chronic inflammatory lung diseases such as asthma and COPD [314].

**Fig. 8:**
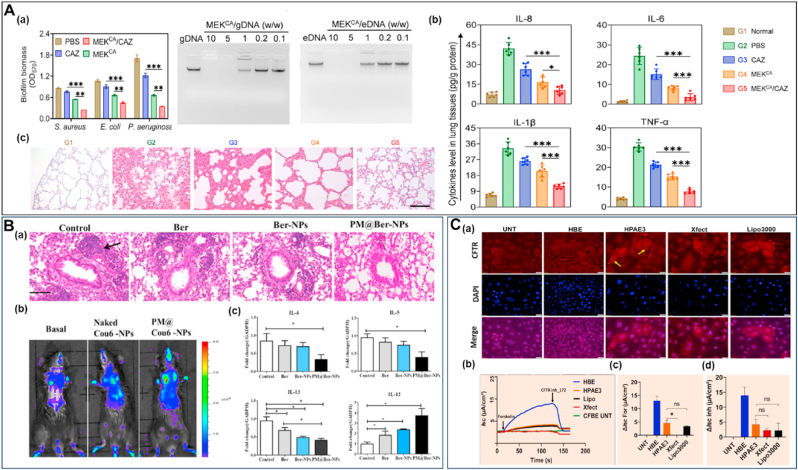
NP-based therapies for respiratory diseases: COPD, Asthma, and CF. **(A)** Inhaled NP-encapsulated IMAM for the treatment of COPD. **(a)** MEK^CA^/CAZ NP significantly attenuated the formation of *P. aeruginosa* biofilms, outperforming both free CAZ and MEK^CA^ NP after a 12-h treatment and scavenged bacterial gDNA and eDNA. **(b)** MEK^CA^/CAZ NP significantly inhibited the release of proinflammatory cytokines in the lung tissue of COPD mice, outperforming MEK^CA^ NP and free CAZ. **(c)** H&E staining of lung tissues revealed attenuation of pathological symptoms following MEKCA/CAZ NP treatment, including improvements in alveolar architecture, reduced alveolar wall thinning, and diminished interstitial edema. Scale bar,100 μm. MEK^CA^: hollow MSNP, MEK^CA^/CAZ: Caz loaded MSNP. Reproduced with permission from Ref. [317] under Creative Commons Attribution 4.0 International License: https://creativecommons.org/licenses/by/4.0/. Copyright from 2024, The American Association for the Advancement of Science. (**B**) Ber loaded biomimetic NP attenuated inflammation in asthma model. **(a)** H&E staining demonstrating that Ber and its nanoparticulate formulations reduce leukocyte infiltration in the airways of the mouse lung, with PM@Ber-NP revealing the most significant beneficial effects in reducing airway inflammation in asthma. Scale bar, 100 μm. **(b)** Cou6-labeled PM@PLGA NP (PM@Cou6-NP) demonstrating enhanced targeting ability to lungs compared to the Cou6-labeled PLGA NP (naked Cou6-NP). **(c)** Ber and its NP reduced Th2 type pro-inflammatory cytokine secretion, but increased anti-inflammatory IL-12 secretion which antagonised Th2-cell responses, with PM@Ber-NP featuring the largest effect. Reproduced with permission from Ref. [336] under Creative Commons Attribution 4.0 International License: https://creativecommons.org/licenses/by/4.0/. Copyright from 2021, Frontiers Media SA. (**C)** Poly β-amino ester/CpG-depleted CFTR plasmid NP for CFTR function restoration in lung CF disease. **(a)** Immunocytochemistry demonstrated the restoration of CFTR protein localized in the cell membrane 48 h after transfection with HPAE3/CpG-depleted CFTR plasmid complexes. **(b)** The transepithelial chloride short-circuit current (I_sc_) was assessed using the chamber assay in response to the CFTR stimulator and inhibitor. **(c)** After stimulation with forskolin, an increase in I_sc_ (ΔI_sc_) was observed with HPAE3/CpG-depleted CFTR plasmid complexes, until CFTR channel inhibition was triggered. **(d)** Following the addition of CFTR inhibitor called CFTRinh_172, a change in ΔI_sc_ (from 131 to 166 ms) was observed in the apical monolayer following transfection with HPAE3/CpG-depleted CFTR plasmid complexes. Reproduced with permission from Ref. [347] under Creative Commons Attribution 4.0 International License: https://creativecommons.org/licenses/by/4.0/. Copyright from 2024, Elsevier.

COPD exacerbations, which are often triggered by bacterial infection results in inflammation induced by bacteria-secreted eDNA and further airway damage [315]. Mucus layer thickening in such conditions promotes clustering of bacteria at the bottom, resulting in biofilm formation [316]. In light of this, mucus and biofilm dual-penetrating immune-antimicrobials present a significant strategy to combat acute COPD exacerbations. Hollow MSNP gated by a transformable polypeptide that owns both positively and negatively charges were loaded with ceftazidime. At neutral pH, the polypeptides exhibited a negative charge and adopted random-coiled conformation, preventing ceftazidime leakage by masking the pores and facilitating the penetration of bronchial mucus and biofilms. In the mildly acidic biofilm environment, the polypeptides were activated, transforming into positively charged α helices that disrupted bacterial membranes and facilitated ceftazidime release and effectively eliminated colonized bacteria (Fig. 8A) [317]**.** While pulmonary delivery of MSNP was not associated with severe toxicity due to their degradation into orthosilicic acid and subsequent renal and hepatobiliary excretion [318], the impact of polypeptide decoration on the biosafety and clearance of MSNP warrants further investigation.

### 7.2 Asthma

Asthma is another chronic inflammatory airway disease characterized by reversible airway obstruction and bronchial hyper-responsiveness [319,320]. Recent research has focused on developing NP formulations of established asthma medications to improve their efficacy while minimizing side effects [321]. Budesonide, a nonhalogenated glucocorticoid, has emerged as a promising candidate for managing asthma, allergic rhinitis, and esophagitis due to its high anti-inflammatory potency [322]. However, the low water solubility of budesonide poses challenges for its inhalation delivery, such as mucociliary clearance and a short residence time of the formulation in the nasal cavity or airways [323]. *In vivo* studies in an OVA-induced asthmatic mouse model showed that budesonide loaded lipid NP extended accumulation of drugs in the lungs, reduced airway inflammation, and improved asthma outcomes compared to free budesonide [321]. To address the short residence time of corticosteroids in the lungs, FcBP-functionalized PEG NP were developed for dexamethasone delivery. These NP were constructed with PEG-coated PLGA-lipid cores decorated with FcBP, a peptide that binds to the neonatal Fc receptor, which enables receptor-mediated transport across the epithelium while avoiding mucus entrapment. Enhanced mucus penetration, cellular uptake, transepithelial transport, and pulmonary distribution were exhibited by the NP. When loaded with dexamethasone, improved pulmonary retention and therapeutic efficacy were demonstrated in asthma mice, with a significant reduction in inflammatory cytokines being observed [324]. Solid lipid NP formulation incorporating rhynchophylline, an alkaloid from the medicinal plant Uncaria, showed augmented lung retention, leading to reduced inflammation of airways and oxidative stress in murine models of allergic asthma. The mechanism involved inhibition of the p38 signaling pathway, a key player in inflammatory responses [325]. In a different approach targeting gene expression, lipid NP were used to deliver siRNA. These NP containing siRNA that targets intercellular adhesion molecule-1 (ICAM-1), notably improved cellular uptake and gene silencing efficiency in human airway epithelial cells (AEC) that express ICAM-1 *in vitro*. In ovalbumin-challenged asthmatic mice, AEC-specific delivery was exhibited by these NP, which effectively downregulated thymic stromal expression of lymphopoietin, alleviated infiltration of inflammatory cells, and reduced the secretion of proinflammatory cytokines (IL-4 and IL-13) and production of mucus [326].

To enhance drug retention at the mucosal surface, mucoadhesive nanocarriers such as chitosan NP have shown particular promise. The positive charge on chitosan NP enhances their efficacy in asthma treatment by promoting mucoadhesion to the negatively charged mucus layer, increasing drug residence time in the lungs. Additionally, this charge facilitates improved cellular uptake and the transient opening of the tight junctions between epithelial cells, allowing for better drug delivery and potential modulation of the immune response [327]. Studies have demonstrated that chitosan NP can significantly reduce airway hyperresponsiveness, total lymphocyte and eosinophil counts in BALF, and pro-inflammatory IL-4 and IL-5 levels in lung tissue, demonstrating attenuation of inflammation in asthmatic mice [328]. The internasal delivery of recombinant IL-17RC (rIL-17RC) was achieved using chitosan NP which promoted inhibition of mucus secretion, reduction of airway inflammatory cell infiltration and pro-inflammatory cytokines in BSLF [329]. Further innovations in chitosan-based NP have led to more specialized applications. For instance, chitosan nanogels grafted with arginine, cross-linked with tris(2-carboxyethyl) phosphine were developed as a mucolytic agent to address mucus plugging in asthma. These nanogels inhibited mucin aggregation *in vitro* through ionic interactions and disulfide bond breakage, with good cytocompatibility up to 5 mg/mL. In an allergic asthma mouse model, nebulized nanogels reduced mucus obstruction and airway inflammation, showing promise for treating muco-obstructive diseases such as asthma [330].

Heparin encapsulated in chitosan and cyclodextrin NP can interact with mast cells to reduce inflammation and airway hyper-responsiveness in asthma models [331]. These NP are used for their ability to enhance drug delivery by facilitating intracellular penetration and reducing cellular toxicity [332]. Heparin exhibited anti-inflammatory effects in asthma models and inhibited mast cell degranulation through interaction with the intracellular receptor inositol trisphosphate (IP3), and possesses mucolytic properties, potentially improving lung function. It also displayed antioxidant effects and may help restore corticosteroid effectiveness in severe asthma, suggesting its potential as an add-on therapy for asthma management [333]. Several studies have demonstrated the anti-asthmatic activity of inhaled heparin, which is independent of its anti-coagulant activity [334,335]. Platelet membrane-coated PEG/PLGA NP (PM@Ber-NP) have been investigated for the targeted delivery of berberine (Ber), a natural anti-inflammatory compound, to inflamed lung tissue. This approach combined the anti-inflammatory properties of Ber with the targeting capabilities of platelet membranes. In a mouse model of house dust mite-induced asthma, PM@Ber-NP demonstrated enhanced retention in inflammatory lung tissue and significantly reduced lung inflammation compared to free Ber. The therapeutic effect was primarily attributed to the regulation of the Th1/Th2 balance, with increased expression of the anti-inflammatory cytokine IL-12 [336] (Fig. 8B).

There has indeed been increasing research interest in ECV as NP carriers for drug delivery in recent years. These naturally occurring nanostructures, including microvesicles and nanoscale exosomes, can transport various proteins and nucleic acids beneficial for managing asthma [337]. Building on the potential of EVs, studies have also explored hybrid NP systems. One example is the use of exosome membrane-coated PLGA NP to deliver Dnmt3aos smart silencer. This approach has revealed effectiveness in targeting M2 macrophages and reducing lung inflammation in allergic asthma mice. These advancements highlight the versatility of EV-based NP in developing targeted therapies for asthma [338].

Some NP possess inherent therapeutic properties beneficial for asthma treatment. For instance, a recent study by Ebeed et al. revealed that polysaccharide beta-glucan NP significantly decreased inflammation of airways, oxidative stress, DNA damage, and ferroptosis in allergic asthma murine model. The study also demonstrated that beta-glucan NP enhanced antioxidant activity and reduced iron deposition, highlighting their potential as a treatment option for allergic asthma [339]. The antioxidant properties of cerium oxide NP were demonstrated through their ability to reduce ROS and DNA damage in lung epithelial cells [340]. Similarly, AuNP and AgNP exhibited anti-oxidant and anti-inflammatory effects in asthma models, thus therapeutic potential for asthma treatment [9].

### 7.3 Cystic fibrosis (CF)

Nanostructures have also shown great promise in the management of CF. CF stands out as a uniquely challenging respiratory disorder, distinguishing itself from conditions like COPD and asthma. Unlike these primarily respiratory diseases that often develop later in life, CF is a genetic disorder resulting from mutations in the CFTR gene. This mutation leads to the production of thick, sticky mucus that obstructs airways, predisposes individuals to chronic bacterial infections, and impacts multiple organ systems from birth [25]. The genetic nature of CF necessitates approaches that can deliver gene therapies or CFTR modulators to correct the underlying defect by enhancing chloride transport in affected cells and improving the function of the defective CFTR protein. The thicker, more viscous mucus in CF poses also greater challenge for NP penetration compared to COPD or asthma. Moreover, CF patients' susceptibility to chronic bacterial infections, particularly *P. aeruginosa*, demands NP designs capable of penetrating bacterial biofilms. The multi-organ involvement and lifelong nature of CF further complicate treatment strategies, requiring consideration of systemic delivery and long-term safety of NP-based therapies [341]. Despite these challenges, significant progress has been made. A triple combination therapy, consisting of elexacaftor, tezacaftor, and ivacaftor, is available for the treatment of CF and has the potential to benefit up to 90 % of individuals affected by the condition. This therapy is currently approved for adults and children aged 2 and older who have specific CFTR mutations [26]. Nasal application in CFTR knockout mice resulted in a recovery of CFTR-mediated chloride secretion for at least 14 days, peaking at 55 % of healthy chloride efflux by day 3 post-transfection, offering potential for correcting the underlying genetic defect [342]. In a phase 1/2 clinical study, inhaled CFTR mRNA (MRT5005) was delivered *via* lipid NP to adults with CF, demonstrating general safety and tolerability, although some subjects experienced mild to moderate fever and hypersensitivity reactions [343]. Additionally, non-viral vectors such as compacted DNA NP have demonstrated safety and partial reconstitution of CFTR function when administered nasally [344]. These NP-based platforms are designed to penetrate the mucus barrier, improve drug bioavailability, and minimize systemic side effects, making them a promising avenue for more effective CF therapies [345].

PLGA NP loaded with antioxidant, glutathione sulfhydryl GSH, and NaHCO_3_ demonstrated, like in the case of other lung diseases, sustained release characteristics, increased apical surface pH, and enhanced mucociliary transport without cellular toxicity in CF epithelium, which are advantageous for CF. *In vivo* tests in rabbits showed no adverse effects, with NP successfully penetrating mucus, indicating their potential as a treatment for CF and other respiratory diseases characterized by mucus obstruction [346]. A polymer-based gene replacement therapy developed by Qiu et al. for lung CF involved screening of various poly(β-amino esters) (PAE) to create non-viral delivery systems that were utilized for restoring CFTR function. The resulting highly branched PAE NP, combined with CFTR plasmid depleted of CpG, demonstrated enhanced gene expression of CFTR and biocompatibility in lung epithelial cells, successfully restoring functional CFTR protein production and showing promise as an efficient and safe treatment for CF patients (Fig. 8C) [347].

Fig. 8 demonstrates the versatility of NP in targeting COPD, asthma, and CF such as MSNP loaded with ceftazidime in the case of COPD [317], berberine-loaded biomimetic NP for asthma treatment [336] or poly β-amino ester/CpG-depleted CFTR plasmid NP for CF [347]. Table 9 highlights the advancements of nanostructures in treating common respiratory diseases such as COPD, asthma, and CF. These findings underscore the potential of nanotechnology-based therapies to overcome treatment limitations and improve targeted disease management.

**Table 9:** NP-based approaches to combat other common respiratory disorders such as asthma, COPD and CF.

NP	Respiratory disorder	Route of administration	Therapeutic functions	Ref.
Curcumin loaded solid lipid NP	Asthma	Intraperitoneal injection	Anti-inflammatory, increase lung and liver distribution, suppress airway hyper-responsiveness and inflammatory cell infiltration, inhibit T-helper-2 cytokine expression	[348]
A20 (ubiquitin E3 ligase) loaded PLGA-ovalbumin	Allergic asthma	Intranasal	Suppress Th2 inflammatory response, promote generation of Treg cells and anti-inflammatory IL-10 in airway tissues, inhibit allergic asthma responses, restore immune homeostasis	[349]
Andrographolide loaded PLGA	Asthma	Oral, inhalation	Reduce inflammation by suppressing NF-κβ signaling more effective by pulmonary route than oral	[350]
Baicalein loaded chitosan	Asthma	Inhalation	Decrease mucus secretion and inflammation, control immune-allergo-inflammatory responses	[351]
PEGylated and citrate/tannic-acid-coated Au NP	Asthma	Intranasal	Increased uptake in asthmatic mice, inhibit inflammatory infiltrates and airway hyperreactivity	[352]
Au NP	Glucocorticoid-resistant asthma	Nebulization	Inhibit airway hyperreactivity, infiltration of eosinophils and neutrophils, production of mucus, and generation of pro-inflammatory cytokine. Improve oxidative stress markers by preserving NRF2 and HDAC2 levels	[353]
PEGylated dextran SPION conjugated with anti-IL4Rα	Asthma	Intranasal	Reduce lung inflammation by decreasing pro-inflammatory cytokine expression, reducing lymphocytes, neutrophils, and eosinophils counts, and deactivating CD4 and CD8 T cells, 1-week post-treatment improved immunosuppressive effects compared to free anti-IL4Rα antibodies	[354]
PLGA-PEG conjugated with ibuprofen	COPD	Intranasal	Targeted delivery of ibuprofen to neutrophils, effective in transporting and releasing the drug to specific inflammatory cells, control neutrophilic inflammation	[355]
SPIO conjugated with CD86 and CD206	COPD	Instillation	Biocompatible for lung administration, facilitate imaging of inflammation	[356]
PEG liposomes loaded with beclomethasone dipropionate	COPD	Inhalation	Efficient mucus penetration and good uptake by airway epithelial cells	[357]
Lipid/polymer loaded with rapamycin	COPD	Inhalation	Effective penetration into bronchial mucus, optimal nebulization performance, AM uptake	[358]
Curcumin loaded PLGA	CF	Oral delivery	Improved processing of DeltaF508 CFTR, enhanced therapeutic effects compared to non-encapsulated curcumin in CF mouse models	[359]
Lipid NP loaded with αmutENaC	CF	Intranasal	Inhibit endogenous heterotrimeric ENaC (epithelial sodium channel) by introducing inactivating mutant ENaC mRNA, increase airway surface liquid height in CF airway cells, reduce mucus dehydration	[360]
Lipid NP loaded with lumacaftor and ivacaftor	CF	Inhalation	Lumacaftor corrects CFTR protein mutation and ivacaftor enhances CFTR channel function	[361]
Iloprost loaded PLGA	CF	Inhalation	Anti-inflammatory effect with attenuation of pro-inflammatory cytokine expression (IL-1β, TNF-α, IL-6, IL-8)	[362]
Nintedanib loaded solid lipid NP	Idiopathic pulmonary fibrosis	Oral	Attenuate lung fibrosis by inhibiting ECM remodeling and collagen deposition	[363]
siRNA loaded lipid NP	Pulmonary fibrosis	Oropharyngeal aspiration	Inhibit expression of heat shock protein 47 and collagen deposition, downregulate 11 fibrosis-relevant genes by approximately 4.5-fold post-treatment, reduce myofibroblast levels	[364]

## 8 Conclusions and future perspectives

This review highlights that, despite significant advancements and developments in the treatment of respiratory diseases, they continue to be a major global health concern. Nanopaticles (NP)- and nanorobots (NR)-based therapeutics for lung diseases enable targeted drug delivery by utilizing passive mechanisms like the EPR effect, as well as active targeting through ligand-based site-specific binding. These strategies enhance therapeutic efficacy while minimizing off-target toxicity. Additionally, they serve as promising alternatives to treatment of lung diseases with antibodies [365,366]. The enormous potential of therapeutic monoclonal antibodies (mAb) has been revealed over more than 20 years, with a handful of mAb having received regulatory approval since 1986 [365]. The positive role of growth factor receptor monoclonal antibodies such as those targeting transforming growth factor-beta (TGF-beta), a major player in producing bronchopulmonary dysplasia [367,368] or pamrevlumab (FG-3019), a human recombinant monoclonal antibody against the connective tissue growth factor, a central mediator in the process of fibrosis, has been underlined in several studies [369]. The same is valid in the case of using anti-vascular endothelial growth factor antibodies such as bevacizumab, employed for the treatment of lung cancer alone or in combination with chemotherapy [370]. While monoclonal antibody therapy has several benefits, including target treatment, lower risk of side effects compared to conventional treatments such as chemotherapy or radiation therapy and being fast acting with therapeutic effects observed typically within a few weeks, potential limitations challenge their application. Next to limited availability and high production costs, monoclonal antibodies can cause side effects (e.g. cardiac toxicity, first-dose toxicity related to rapid lysis of malignant cells, skin eruptions, etc). These factors may make it difficult to achieve optimal results and require a close management of side effects for patients receiving monoclonal antibody therapy. Due to their large size, antibodies cannot be eliminated through the kidney and often remain in the bloodstream and interact with the tumor cells inefficiently. This makes the exploration of alternative approach such as NP and NR-based therapeutics of pending interest.

Diverse materials have been explored, each presenting unique advantages and limitations. Polymeric NP enable controlled drug release and can help overcome mucosal barriers. Drug-loaded liposomes and lipid NP enhance lung accumulation time and improve drug retention due to their membrane-like properties. Magnetic NP facilitate targeted therapy through external guidance, while metal NP excel in imaging and photothermal applications. Carbon NP show potential for drug delivery, but require thorough toxicity evaluation. BioNR integrate biological and synthetic components to enhance targeting, though their safety profile still needs to be fully assessed.

While the problem of targeted transport of NP and NR to tumor cells and infection sites has been largely solved by the ability to add target molecules to the outside of these nanostructures, as validated in *in vitro* studies, the next years will require a complex task focusing on their investigation inside the human body. In most therapeutics, most of the drugs, including NP and NR, are formulated for systemic intravenous or oral administration. These administration routes are associated with reduced bioavailability at the target site and excretion of the nanostructures in the kidney and the phagocytic system. The development of inhalable nanostructures holds great promise in this field. Drug degradation during formulation and nasal delivery *via* sprays, and inhalators remain of concern, particularly for sensitive drugs. Another challenge lies in ensuring the stability of NP during production, storage, and delivery. NP are prone to aggregation, which can reduce their efficacy and alter their pharmacokinetics. Moreover, scaling up production from laboratory to industrial levels while maintaining consistency and efficacy remains a significant barrier to widespread clinical application. For diseases that affect the deeper lung tissues, such as CF or lung cancer, ensuring that nanostructures reach the alveoli is particularly important, but current delivery methods often result in uneven distribution. Ongoing research into bioNR show partial promise in reaching difficult-to-reach areas within the respiratory system in an efficient and patient-friendly manner. However, bioNR are still in the experimental and even conceptual phase, with several hurdles remaining before such therapies become routine in clinical practice. Issues like toxicity, reproducibility and upscaling need to be addressed still. The question of long-term safety, particularly regarding their potential to induce inflammation or toxicity and the interrogation of the clearing mechanisms from the lungs has still to be explored. Furthermore, regulatory hurdles include rigorous safety evaluations to address concerns about long-term toxicity, immunogenicity, and potential off-target effects of these nanostructures exist. Ensuring reproducibility, scalability, and compliance with Good Manufacturing Practices (GMP) for large-scale production remains a significant barrier to clinical adoption. Ethical concerns also arise regarding *in vivo* testing on animal models. The potential for unintended consequences—such as uncontrolled bacterial proliferation or adverse immune responses—underscores the importance of thorough preclinical and clinical testing.

While animal models have shown encouraging results, translating these findings to human clinical trials has been slow due to differences in lung physiology between species. Another limitation is the variability in patient responses. Factors, such as lung anatomy, disease progression and patient breathing patterns, can affect how well NP are deposited and retained in the lungs. This variability makes it difficult to develop a one-size-fits-all solution and suggests the need for more personalized approaches in nanomedicine. One of the most significant issues is the inability to assess the treatment's efficacy during administration. This could be addressed by using a multi-functional NP that can carry both therapeutic and imaging modalities, allowing real-time monitoring of drug delivery and efficacy. The use of targeted ligands like peptides or antibodies will be critical in increasing their lung deposition while decreasing off-target effects. Future advances may involve combining immunotherapy or gene therapy with NP platforms to address complex lung diseases like cancer and fibrosis. This will require continued innovation in the design and fabrication of bioengineered materials that are reproducible, scalable, and cost-effective. Advances in the fabrication of bioengineered materials could solve present limitations such as reproducibility in production and scalability. Needless to say, robust, reproducible, and cost-effective production of multifunctional nanomaterials would be vital for the advancement of the clinic and beyond. In summary, although the utilization of nanomedicine for treating lung disorders faces several challenges, advancement in current research suggests that, in due course, more effective, precisely targeted, and even personalized treatment may be achieved. Ongoing innovation in the design of nanostructures, a deeper understanding of lung delivery mechanisms, and clinical translation will play an essential role in unlocking the full potential of this technology in the years to come.

## CRediT authorship contribution statement

**Meekha George:** Writing – original draft, Conceptualization. **Rabah Boukherroub:** Writing – review & editing. **Amitav Sanyal:** Writing – review & editing. **Sabine Szunerits:** Writing – review & editing, Funding acquisition, Conceptualization.

## Funding

The project is funded by the 10.13039/501100007601Horizon 2020 framework program of the 10.13039/501100000780European Union under grant agreement No. 101129095 (LungCare).

## Declaration of competing interest

The authors declare that they have no known competing financial interests or personal relationships that could have appeared to influence the work reported in this paper.

## Data Availability

No data was used for the research described in the article.
